# Extracellular vesicles in cancer´s communication: messages we can read and how to answer

**DOI:** 10.1186/s12943-025-02282-1

**Published:** 2025-03-19

**Authors:** Alena Semeradtova, Michaela Liegertova, Regina Herma, Magdalena Capkova, Chiara Brignole, Genny Del Zotto

**Affiliations:** 1 Institute of Photonics and Electronics of the CAS, Chaberská 1014/57, Prague, 182 51 Czech Republic; 2 Centre for Nanomaterials and Biotechnology, Faculty of Science, Jan Evangelista Purkyně University in Ústí Nad Labem, Pasteurova 3632/15, Ústí Nad Labem, 40096 Czech Republic; 3 Laboratory of Experimental Therapies in Oncology, IRCCS Istituto Giannina Gaslini, Via G. Gaslini 5, 16147 Genoa, Italy; 4 Core Facilities, Department of Research and Diagnostics, IRCCS Istituto Giannina Gaslini, 16147 Genoa, Italy

**Keywords:** Extracellular vesicles, Cancer, Tumor microenvironment, Biomarkers, Liquid biopsy, Metastasis, Immune evasion, Targeted therapy

## Abstract

Extracellular vesicles (EVs) are emerging as critical mediators of intercellular communication in the tumor microenvironment (TME), profoundly influencing cancer progression. These nano-sized vesicles, released by both tumor and stromal cells, carry a diverse cargo of proteins, nucleic acids, and lipids, reflecting the dynamic cellular landscape and mediating intricate interactions between cells. This review provides a comprehensive overview of the biogenesis, composition, and functional roles of EVs in cancer, highlighting their significance in both basic research and clinical applications. We discuss how cancer cells manipulate EV biogenesis pathways to produce vesicles enriched with pro-tumorigenic molecules, explore the specific contributions of EVs to key hallmarks of cancer, such as angiogenesis, metastasis, and immune evasion, emphasizing their role in shaping TME and driving therapeutic resistance. Concurrently, we submit recent knowledge on how the cargo of EVs can serve as a valuable source of biomarkers for minimally invasive liquid biopsies, and its therapeutic potential, particularly as targeted drug delivery vehicles and immunomodulatory agents, showcasing their promise for enhancing the efficacy and safety of cancer treatments. By deciphering the intricate messages carried by EVs, we can gain a deeper understanding of cancer biology and develop more effective strategies for early detection, targeted therapy, and immunotherapy, paving the way for a new era of personalized and precise cancer medicine with the potential to significantly improve patient outcomes.

**Supplementary Information:**

The online version contains supplementary material available at 10.1186/s12943-025-02282-1.

## Introduction

Intercellular communication is a dynamic process that reflects emerging changes in cancer initiation and progression and enables monitoring of those changes via alternation of signals transmitted within cells and their surrounding environment. EVs contain surface molecules targeting their pathways or, together with the bioactive cargo, influencing the function of recipient cells. This evidence supports the notion that EVs play multiple roles in crucial processes that are essential not only for the physiological cell-to-cell communication but also for cancer initiation, progression and dissemination and, therefore, can serve as a very useful source of information about those processes [1].

EVs mirror processes associated with cancer progression, such as hypoxia, chronic inflammation and immune system surveillance and play key roles in tumor escape. They contain information regarding the cell of origin, cancer-related changes inclination, and potential sites of metastasis, as well as the response to treatment, including the possibility of developing resistance to therapy and/or specific features of dormancy [2]. EVs are easily accessible in the body fluids and can therefore provide a harmless source of information about ongoing processes and allow us to monitor the progression of cancer-related transformations, disease progression and response to therapy [3]. Therefore, every identified change in cancer-related EVs composition is a potential biomarker and single pixel of information that finally enables us to portray the ongoing cancer-related transformations and, moreover, can help us to target anticancer therapies more effectively [4, 5].

In this review, we will point to distinct areas where EVs were proven to contribute to the cancer progress with emphasis on the relevant EVs surface molecules and cargo that mirror the ongoing process and therefore can serve as a gadget to complete the informative panel of markers of liquid biopsy and can help to estimate the right defense and therapy. Moreover, our increasing capacity to alter the content of these vesicles is starting to be utilized to create innovative therapies; therefore, finally, we will briefly introduce how EVs can be employed in cancer treatment.

## Categorization of extracellular vesicles

Over the past decades, numerous small particles have been independently discovered and described by various research groups across diverse biological samples [6]*.* Finally, these particles were consolidated under the designation extracellular vesicles, and as our understanding of EVs has grown, it has become increasingly clear that these vesicles represent a diverse and heterogeneous population with distinct characteristics and functions.

The classification of EVs is a complex and challenging task due to their overlapping characteristics and the continual discovery of new subtypes. However, efforts have been spent to establish standard guidelines and nomenclature to facilitate the study and discussion of EVs [7–9]. One common approach to classify EVs is based on their size, with small EVs being defined as those measuring < 100 nm or < 200 nm, and medium/large EVs being those > 200 nm in diameter [8]. A special heterogeneous category of EVs includes extracellular particles measuring below 50 nm [10]. This size-based classification provides a useful framework for characterizing and comparing different EV populations.

**Fig. 1:**
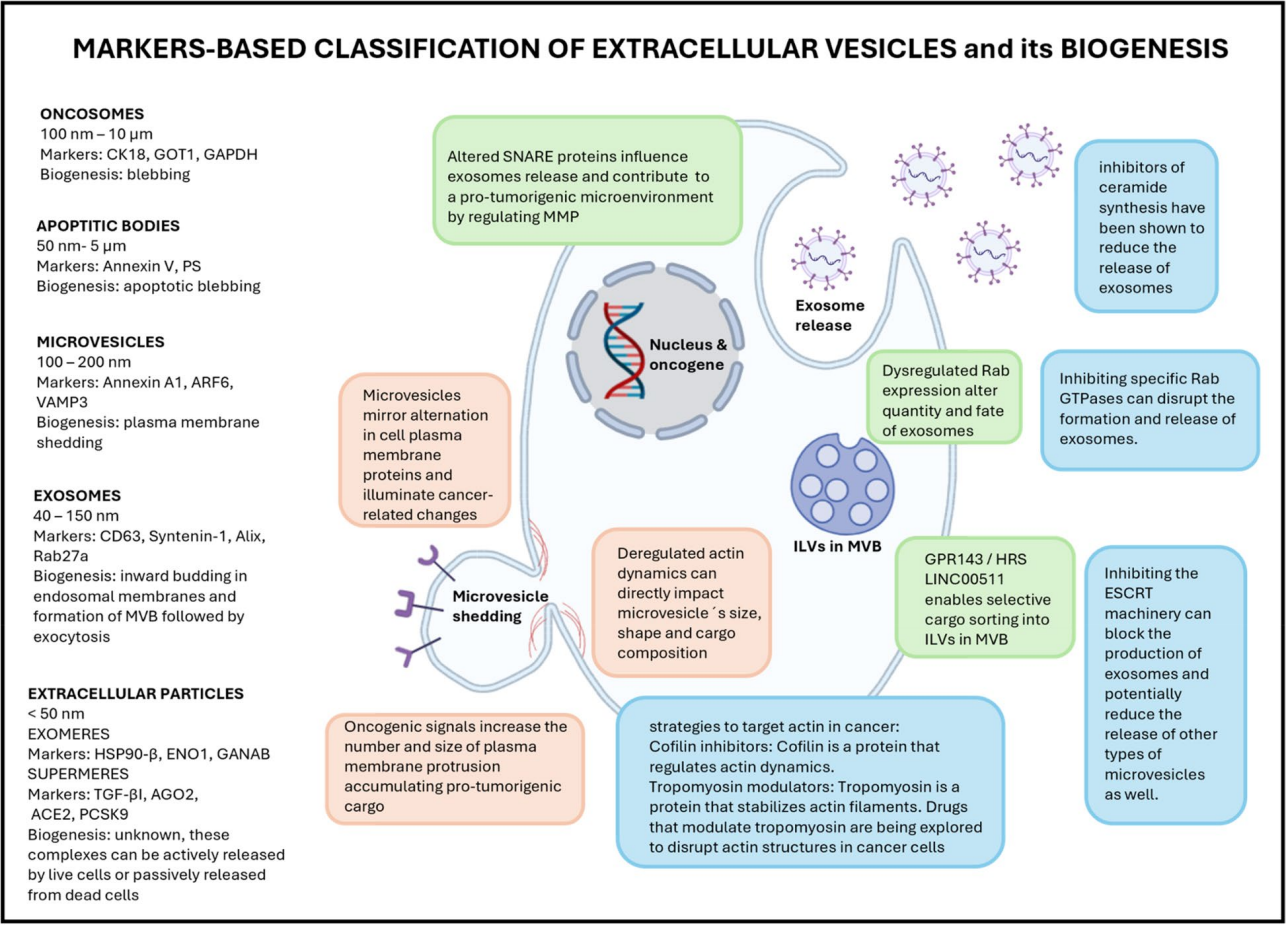
Classification of extracellular vesicles (EVs) and illustration of their biogenesis. EVs are classified based on their biogenesis mechanism concept (e.g., exosomes, microvesicles, apoptotic bodies and oncosomes). Exomeres and supermeres represent a special subtype of EVs themed extracellular particles. However, exomeres are morphologically distinct from supermeres, both types of extracellular particles were described to carry clinically relevant cargo. EVs exhibit several cancer-related quantity and quality modifications pointing to the development of oncogenic processes. On the other hand, vesicles´ biogenesis exhibits promising cancer therapy. Created in BioRender. Čapková, M. (2025) https://BioRender.com/y45t011

In addition to size, EVs can also be classified based on their cellular compartment of origin [11]. Exosomes, which range in size from 40–150 nm, derive from the endosomal membrane and have been extensively studied for their role in intercellular communication. Ectosomes, on the other hand, are shed directly from the cell's plasma membrane and can be further classified into microvesicles (100–200 nm) and small/large oncosomes (100 nm–10 μm), with the latter being exclusively produced by cancer cells. Apoptotic bodies (50 nm–5 μm) represent another major subtype of EVs, generated during the process of programmed cell death through the characteristic membrane blebbing [8]. Newly identified subpopulations of EVs, termed ‘exomeres’ and ‘supermeres,’ are also characterized by a size of ≤ 50 nm. Unlike other EV subtypes, exomeres have been defined as non-membranous nanovesicles, whereas supermeres, though similarly sized, differ both morphologically and structurally from exomeres and exhibit distinct cellular-uptake kinetics compared to small EVs and exomeres [10, 12]. For more details, see Fig. 1.

## Understanding EVs diversity: biogenesis and implications in oncogenesis

Because the various EV subpopulations often overlap in size, isolating them with both high yield and precision remains challenging. This difficulty impedes the study of individual EVs subtypes and underscores the value of strategies that inhibit specific biogenesis pathways. Moreover, suppressing EVs biogenesis has emerged as a promising therapeutic avenue for cancer treatment.

Cancer cells are well recognized to secrete substantially higher quantities of EVs than their nonmalignant counterparts, frequently with altered compositions [13–16]. Both changes in EVs content and increases in secretion rate may contribute to the carcinogenic effects of tumor-derived EVs. While elevated EVs secretion can lead to excessive stimulation of recipient cells, shifts in EV composition—through modifications in EVs subtypes or cargo loading—can induce tumor-promoting changes in the microenvironment. Numerous oncogenes and tumor suppressor genes, in both wild-type and mutant forms, have been implicated in regulating EVs biogenesis [17, 18]. For summarization of cancer-related changes in biogenesis, see Table 1.

**Table 1:** Cancer-related changes in EVs biogenesis

Regulator/Pathway	Normal cells	Cancerous cells	Quantitative outcome	Ref
EV size & types	relatively uniform, ~ 40–150 nm, mostly one population with consistent markers	broad size ranges, heterogenous subpopulations including microvesicles, apoptic bodies, ectosomes	cancer-derived exosomes show bimodal distribution (exosomes ~ 100 nm, microvesicles 200–500 nm)	[19, 20]
Secretion rate	stimuli-dependent intermittent release, relatively low EV counts in circulation	continuous release, relatively high EV counts in circulation	tumor cells produce ~ 10 × more exosomes than normal cells	[21]
Rab27 (A/B)	low-to-moderate expression; active in specialized secretory cells for regulated exosome release	overexpression in many tumors, drives high exosome output; essential for metastasis-supporting EVs; elevation correlates with poor survival	elevation correlates with poor survival	[22, 23]
Rab35	involved in endosomal recycling, not a dominant exosome regulator	upregulation in various cancers, often exhibits oncogenic effect	downregulation via miR-185 => loss of protumorigenic exosome effects; NSCLC: significant increase compared to normal lung epithelium leukemia: facilitates immune evasion	[24, 25]
Rab11	ubiquitous recycling endosome Rab, involved in ILV trafficking in certain contexts, modest impact on exosome release	hyper-activated via Ca^2+^-Munc13-4 pathway, enables rapid MVB maturation and release; in tandem with Rab27 enables continuous exocytosis	acute Ca^2+^ influx increases exosome release fivefold Munc13-4 often elevated in aggressive cancers (late-stage breast cancer, pancreatic tumors)	[22, 26]
SNARE complex (syntaxin-4, SNAP-23, VAMP-7)	expression aligned with secretory needs, participates in vesicle fusion; SNAP-23 usually membrane-bound in lipid rafts	upregulated/activated by oncogenic signals; required for exosome release; SNAP-23 often liberated from rafts by Src	elevation of SNAP-23 correlates with worse prognosis; Src activation of SNAP-23 in cancer => increased EVs production; breast cancer model: VAMP-7 knockout => decreased exosome release and lung metastasis	[27, 28]
ESCRT proteins (Hrs, TSG101, ALIX, etc.)	support ILV formation in a regulated manner	overexpression sustains high ILV biogenesis; needed for exosome production	ovarian carcinoma: TSG101 upregulated in ~ 70% of Ras-driven carcinomas; HeLa tumor model: silencing of Hrs or TSG101 => decreased EVs secretion	[11, 29]

### Exosome biogenesis

Exosomes arise from the endosomal pathway, a complex network initially designed for sorting and degrading cellular components. Cancer cells, however, exploit this system, turning it into a production line for pro-tumorigenic EVs.

The key steps of exosome biogenesis are: (i) Inward Budding: the endosomal membrane invaginates, forming intraluminal vesicles (ILVs) within multivesicular bodies (MVBs). This process, tightly controlled by the Endosomal Sorting Complex Required for Transport (ESCRT) machinery, relies on ubiquitin tagging of target proteins for ILV inclusion [30, 31]. In cancer, oncogenic signaling pathways can directly influence ESCRT activity, leading to the preferential packaging of oncoproteins, growth factors, and immunosuppressive molecules such as RNA molecules (for more information, see Table 2) into exosomes, transforming them into potent vehicles for tumor progression [32]. Lee et al. identified that GPR143 (G-protein coupled receptor 143) interacts with Hrs (an ESCRT-0 subunit) and promotes its association with cargo proteins, which subsequently enables selective protein sorting into intraluminal vesicles in MVB. GPR143 is elevated in multiple cancers, where GPR143-ESCRT pathway promotes the secretion of EVs that carry unique cargo, including integrins and signaling proteins [33]. It was described by Hoshino et al. that Hrs also promoted invadopodia formation and cell invasion in SCC61 and head and neck squamous carcinoma cell lines. Invadopodia are part of MVBs docking and secretion mechanisms and significantly increase the secretion of EVs and metalloproteinases favoring tumor invasion and metastasis [34]. Peng et al. revealed that similarly, a long noncoding RNA LINC00511 is involved in MVB trafficking, exosome secretion, invadopodia formation, and tumor invasion [35].

**Table 2:** Different types of RNA contained in EVs

**messenger RNA (mRNA)** • full-length (up to 5000 bp) or fragments (250-700 nt) o fragments: either stable products of degradation of full-length mRNA (disposal via EVs) or isolated 3’UTRs (molecular ‘sponge’ for regulatory miRNA and translation factors, regulates gene expression via EVs) [36] **circular RNA (circRNA)** • single-stranded, highly stable, both in linear and circular form o ‘sponges’ for miRNA and proteins o scaffolds for transport of miRNA or proteins o may play a vital role in the development of diseases [36, 37] **micro RNA (miRNA)** • ~ 22nt, single-stranded o regulate mRNA expression, usually through interaction with 3’UTRs => translational repression or degradation o most studied RNA cargo in EVs (up to 30 % of total reads in small RNA sequencing) o disease biomarkers, RNA-mediated therapies [38–40] **transfer RNA (tRNA)** • full-length or fragments o seems to act as a regulatory molecule in various cellular processes (cell-to-cell communication, inhibition of translation, stress response, gene expression…) [41] **ribosomal RNA (rRNA)** o function in EVs not well understood [42] **long non-coding RNA (lncRNA)** • > 200 nt, not translated into protein o function in EVs not well understood [36] **small nucleolar RNA (snoRNA)** • not translated into protein, guides chemical modification of other RNAs o function in EVs not well understood [43] **piwi-interacting RNA (piRNA)** • silencing of the transposable elements o function in EVs not well understood [44] **Y RNA** • small non-coding RNA, involved in cellular processes (DNA replication, transcription, translation) o function in EVs not well understood [45] **vault RNA (vtRNA)** • forms a complex with proteins to create vault particles (found in cytoplasm of eukaryotic cells) o function in EVs not well understood [46]	

(ii) MVB Trafficking: Once formed, MVBs must navigate a complex intracellular landscape, avoiding degradation by lysosomes [47–49]. This journey is directed by Rab GTPases, master regulators of membrane trafficking [50–52]. Cancer cells frequently exhibit dysregulation of Rab expression, rather than mutation of Rab genes, altering both the quantity and destination of exosomes [53]. Rab proteins can participate in the activation of oncogenic signaling pathways like mTORC1 or PI3/AKT [54–56]. They can contribute to increased cell migration and invasion because they regulate the trafficking of integrins, which are crucial for cell adhesion and migration [55, 57, 58]. They can also regulate cell migration by interacting with vimentin and Ras-related C3 botulinum toxin substrate 1 [59]. Rab proteins, particularly Rab27 also participate in enhanced exosome secretion and have been implicated in the secretion of EVs containing miRNAs that enhance cancer cell proliferation and migration in gastric cancer [53, 60–62]. Rab proteins are key regulators of cellular processes implicated in cancer development and progression. Targeting these proteins could potentially be a therapeutic strategy for certain cancers.

(iii) Exosome Release: Exosome biogenesis culminates in the fusion of MVBs with the plasma membrane, releasing ILVs as exosomes into the extracellular space. This fusion process, mediated by SNARE proteins, represents another point of manipulation for cancer cells [63–65]. For example, syntaxin 6 and VAMP3 regulate MVB-plasma membrane fusion and exosome release in prostate cancer [66]. Similarly, SNAP23 and VAM3 are required for fusion in hepatocellular carcinoma [67, 68]. Altered SNARE expression also contributes to a pro-tumorigenic microenvironment by regulating matrix metalloproteinases (MMPs) secretion, or syntaxin-3 and 4, promotes integrin trafficking and therefore cell migration, and survival [69–76].

Due to the fact that tumor-derived exosomes are shaping TME homeostasis, possibilities of inhibition of exosome production in cancer and stromal cells were investigated to reduce cancer growth and metastasis [77]. Recently, several inhibitors operating through distinct mechanisms were described, ultimately reducing exosome secretion by blocking the ceramide-modulating inward budding of MVBs and the subsequent release of exosomes from them [78]. Other inhibitors target ATP-sensitive K^+^ channels or ATP-binding cassette transporters. These inhibitors regulate cellular cholesterol and phospholipid concentrations, ultimately inhibiting the release of MVBs and exosomes [79]. Another group of inhibitors targets cytoskeletal organization, which is essential for exosome release as well as for endocytic processes [80]. Another mechanism is targeting the Ras/Raf/ERK1/2 signaling pathway, which is crucial for the ESCRT-dependent exosome biogenesis [81, 82].

### Ectosome biogenesis

Ectosomes, unlike exosomes, bud directly from the plasma membrane, making their content a direct reflection of the cell surface landscape, which is often dramatically altered in cancer. Alterations in membrane proteins (MPs) and their regulated pathways have been established as cancer hallmarks and extensively targeted in clinical applications. Li et al. systematically integrated MP interactions, genomics, and clinical outcomes for helping illuminate cancer-wide atlas and prognostic landscapes in tumor homo/heterogeneity and identifying prognostic biomarkers and druggable targets [83]. As already mentioned, vesicles budding from the plasma membrane of the cell keep the same composition; therefore, this atlas can also be applied in EVs-based liquid biopsy and can bring valuable information comparable with the evaluation of circulating tumor cells.

Ectosome biogenesis can be divided into three consecutive processes: Membrane Protrusion, Budding and Scission. Localized membrane protrusions, like filopodia and microvilli, serve as platforms for ectosome formation. Cancer cells, driven by oncogenic signaling, often display exaggerated membrane dynamics, leading to an increase in the number and size of these protrusions [84–88]. This provides ample space for accumulating pro-tumorigenic cargo, including MMPs, adhesion molecules, and signaling receptors, priming ectosomes for their role in invasion and metastasis [75, 89]. Actin stress fibers, membrane ruffles, lamellipodia, and filopodia are formed as a result of the activation of specific Rho GTPases—Rho, Rac1, and Cdc42—by WASP and WAVE. These actin structures are not merely components of cellular architecture; they are actively involved in the directional motility of cancer cells, a critical process in the invasion of surrounding tissues and the progression to metastasis. These proteins facilitate the dynamic reorganization of the actin cytoskeleton, which in turn allows cancer cells to establish cellular protrusions that are crucial for their interaction with the extracellular matrix and movement. This interaction is particularly crucial for the cells' capacity to degrade barriers, which is a prerequisite for invasive behavior. Additionally, these structures facilitate the cells' ability to navigate intricate extracellular environments, which contributes to their metastatic dissemination. Therefore, these proteins are emerging as potential targets for therapeutic interventions that are designed to reduce cancer metastasis by affecting cell motility and actin dynamics [90, 91]. Finally, the membrane protrusion pinches off, encapsulating cytoplasmic contents within an ectosome. This intricate process also relies on the actin cytoskeleton and cancer-associated proteins (CAPs), which are frequently exploited by cancer cells. Deregulated actin dynamics, a hallmark of tumor progression, can directly impact ectosome size, shape, and cargo composition, further amplifying their pro-tumorigenic potential [92].

There have already been suggested GPR77 or mesothelin neutralizing antibodies that inhibit the promotion of protumorigenic cancer-associated fibroblast (CAFs); however, CAF-targeted clinical trials did not recapitulate the advantageous effect from preclinical models yet [93, 94].

### Apoptotic body biogenesis

Apoptotic bodies, while a consequence of programmed cell death, are not inert debris. They too carry a legacy of the cancer cell, with potential consequences for tumor progression [95, 96]. As cancer cells undergo apoptosis, their membranes undergo dramatic blebbing, forming large protrusions containing fragmented organelles and cytoplasmic contents [97, 98]. These blebs detach, forming large apoptotic bodies. While not actively secreted, they can be taken up by neighboring cells, potentially transferring oncogenic signals, drug resistance factors, or immunosuppressive molecules, even in death contributing to a pro-tumorigenic environment [99, 100].

## Alternation of EVs composition: molecular architects in cancer progression

As already explained, EVs play a multifaceted role in cancer progression, influencing various stages from tumor growth to metastasis and also contribute to the immune system escape. Their diverse composition, reflecting both their biogenesis machinery and the dynamic cellular environment, serves as a molecular fingerprint of the originating cell and a potent tool for intercellular communication. Therefore, it is important to find a way to read those messages, because it can help us to reveal the ongoing process and predict the progression. This chapter delves into the cargo landscape of EVs, highlighting how their composition mirrors cancer-related alterations, communication within tumor and its microenvironment and enables the immune system surveillance. This EVs cargo can serve as a very promising source of information and certain biomarkers can provide a roadmap to monitor the cancer progression and a potent navigation to target the therapy.

### Who is who? Functional consequences of the EVs origin to the cancer development

EV subpopulations in cancer are often challenging to distinguish by size as well as functionally, yet their origin and uptake determine their specialization [101]. Exosomes, formed within multivesicular bodies and released via plasma membrane fusion, carry specific cargo like tetraspanins and small RNAs. Ectosomes bud directly from the cell surface, encapsulating cytosolic and membrane proteins. Exosomes are enriched in endosomal markers (Alix, TSG101, CD63), while endosomes contain more plasma membrane components (integrins, Annexin A1) [102]. Consequently, exosomes often deliver signaling proteins or genetic regulators, reprogramming gene expression, whereas endosomes present surface-bound molecules, directly triggering receptors [102–104].

Exosome uptake typically involves endocytosis or membrane fusion, activating intracellular signaling. For example, exosomal integrins activate the Src kinase pathway in lung fibroblasts, promoting metastasis [105]. Similarly, tumor exosomes carrying oncogenic KRAS or EGFRvIII can drive proliferation [101]. Ectosomes, conversely, often engage surface pathways. FasL-bearing microvesicles initiate apoptosis in T cells [103], and tissue factor TF-expressing microvesicles trigger coagulation on endothelial surfaces [104].

EV subtype interactions with target cells also differ. Small exosomes (100 nm) can circulate widely, even crossing barriers, due to their size and surface proteins, enabling selective activation of distant cells [105]. Larger microvesicles and especially large oncosomes have shorter ranges, often being cleared by phagocytes. However, they readily interact with nearby cells expressing receptors for their ligands, such as immune or endothelial cells [104].

EV release and action timing also vary. Exosome secretion is relatively continuous and upregulated by stress factors (hypoxia, acidosis), accumulating early in tumor development to pre-condition distant sites for metastasis [105]. Microvesicle shedding, triggered by acute stimuli (e.g., calcium spikes, shear stress, RhoA/ROCK signaling during amoeboid transition) [106], is more immediate and transient. For example, TF^+^ microvesicles rapidly activate endothelium [104], and FasL^+^ vesicles induce T-cell apoptosis within minutes of contact [103]. While microvesicles are quickly cleared, exosomes can persist longer, exerting prolonged influence (e.g., sustaining fibroblast activation or long-term reprogramming of bone marrow progenitors). In vivo, large oncosomes correlate with tumor stage and aggressiveness [106], while exosomes are abundant in early-stage cancers, contributing to immune evasion and niche formation [105, 107].

### Signs of cancer-related changes

EVs, carrying a diverse array of proteins and RNAs, offer valuable insights into cancer progression. They serve as biomarkers, reflecting ongoing oncogenic changes and facilitating intercellular communication. Proteomic studies have identified reliable surface pan-EV markers (CD9, HSPA8, HSP90AB1, ACTB, MSN, and RAP1B) and tumor-specific markers (thrombospondin-2, tenascin C, and VCAN), aiding in the distinction between cancer-derived and non-cancerous EVs [108]. Cell origin and tissue-alteration markers have also been extensively studied in various cancer types, such as breast cancer, ovarian cancer, non-small cell lung cancer, and bladder cancer [109–114]. EVs diagnostic potential goes hand in hand with their crucial role in intercellular communication. They carry signaling molecules that can activate surface receptors or be internalized by recipient cells, influencing cellular behavior. For example, connexins, particularly Cx43, have been shown to regulate EV uptake [115].

Inflammation, a frequent companion of cancer, is influenced by EVs, and EVs also bring the signs of inflammation on their surface. Tumor-derived EVs often contain inflammatory cytokines and can activate the NF-κB pathway in recipient cells, leading to increased expression of pro-inflammatory mediators [116, 117]. Additionally, EVs can carry miRNAs like miR-21 and miR-181b-1, further amplifying the inflammatory response and contributing to cancer progression [118]. Damage-associated molecular patterns (DAMPs) can also be associated with EVs and play a role in cancer development. Hoshino et al. have identified DAMPs enriched in tumor-derived EVs, including S100A13, basigin, galectin 9, biglycan, and integrin α5 and αX [2].

Soluble molecules within EVs constitute a diverse class of proteins or RNAs found in the lumen. Enclosed within the EV membrane, these molecules are shielded from the external environment, rendering them stable and resistant to degradation. These molecules act as messengers, pivotal in intercellular communication by transmitting signals from the donor to the recipient cell. This internal cargo of EVs, particularly RNAs, holds significant promise as biomarkers and therapeutic targets (see Table 2: Different Types of RNA Contained in EVs). These RNA molecules can regulate gene expression and function in recipient cells, influencing cancer proliferation and progression. They affect this process in both directions to accelerate and promote it as well as they may contribute to its suppression. Therefore, multiple RNAs, lipids, and proteins are suggested as cancer biomarkers, while others may hold potential to be employed in cancer treatment (see Supplementary Table 1 for RNAs and Supplementary Table 2 for proteins and lipids).

### EVs contribution to angiogenesis

Increased multiplication of tumor cells is usually accompanied by a lack of nutrients and oxygen. For further tumor growth, angiogenesis, the formation of new blood vessels, is a critical process. Vascular endothelial growth factor (VEGF), a proangiogenic factor secreted by both endothelial and tumor cells, is a primary driver of this process [119–121]. EVs containing VEGF likely play a significant role in early tumor angiogenesis [122]. Studies have shown that EVs can activate a specific form of VEGF called VEGF90K [123, 124]. The presence of heat shock protein 90 (Hsp90) near exosomal VEGF has been demonstrated to reduce the effectiveness of bevacizumab, a monoclonal antibody targeting VEGF-A, and contribute to the resistance of angiogenesis-targeted treatments [125]. Enrichment of Hsp90/p‐IKKα/β complex in hypoxic melanoma-derived EVs can activate the IKK/IκB/NF‐κB signaling pathway, leading to increased expression of CXCL1 and promoting melanoma angiogenesis and progression [126]. Malignant transformation of prostate epithelial cells often entails a notable shift in the intracellular localization of galectin-3 (Gal-3) [127]. Under normal conditions, Gal-3 is mainly sequestered within the nucleus, where it exerts anti-apoptotic functions. In contrast, prostate cancer cells typically show a cytoplasmic redistribution of Gal-3. This change fosters tumor growth, promotes angiogenesis, and confers resistance to therapy [128]. Notably, elevated cytoplasmic Gal-3 can be actively packaged into EVs, where it may serve as a prognostic biomarker of disease progression [129].

Other proteins found in EVs, such as carbonic anhydrase 9 [130], annexin II [131, 132], and WNT5 [133], also contribute to angiogenesis and represent potential biomarkers as well as targets for antiangiogenic therapy. EVs derived from pancreatic cancer have been found to activate the PI3K/Akt or MAPK signaling pathways [134, 135]. Pancreatic cancer-derived EVs were shown to contain miR5703 downregulating the CMTM4 or miR4465 and miR616-3P repressing PTEN and activating the Akt [136, 137]. Targeting mTOR, a component of the PI3K/AKT pathway, is a common approach to inhibit tumor growth and angiogenesis [138, 139]. Lu et al. demonstrated that miR-338 can inhibit proliferation and autophagy by targeting ATF2 via the PI3K/AKT/mTOR pathway in cervical cancer cells, suggesting its potential as a novel therapeutic target [140]. The list of other RNA molecules promoting and suppressing angiogenesis, and therefore presenting potential biomarkers and components of targeted therapy, is presented in Supplementary Table 3.

### EVs-mediated epithelial-to-mesenchymal transition, metastasis and organotropism

The transition of cancer cells from a benign to a metastatic state is often characterized by a shift in cellular phenotype, known as epithelial-mesenchymal transition (EMT). This is another key point determining the cancer progression and has enormous importance for monitoring of cancer development. This shift is usually diagnosed by the downregulation of epithelial markers like E-cadherin and the upregulation of mesenchymal markers like N-cadherin and vimentin on cells [141]. As explained above, endosomes keep the signs of parental cells on their surface; therefore, those changes can be determinants on tumor-derived EVs.

Hypoxia, low oxygen levels, is a common condition within tumors and plays a significant role in driving EMT and metastatic spread. While hypoxia influences the cargo of EVs and their properties, a universal EV marker specific to hypoxic conditions has not yet been identified. On the other hand, a set of secondary signs of experiencing hypoxia can be employed to monitor cancer development. Several EMT-inducers, including TGFβ, HIF-α, β-catenin, caveolin-1, and vimentin, have been found within EVs produced by solid tumors under hypoxic conditions [142–145]. Additionally, stromal cells within TME can facilitate EMT, invasion, and metastasis. For example, bone marrow-derived mesenchymal stem cells and their hypoxia-secreted EVs have been shown to transfer specific miRNAs (miR-193a-3p, miR-120-3p, and miR-5100) to surrounding lung cancer cells, activating the STAT3 signaling pathway and inducing an EMT phenotype [146].

Beyond their role in EMT, EVs secreted by hypoxic cells can also contribute to the increased mobility and invasiveness of cancer cells. Kumar et al. demonstrated that these EVs can activate the production of MMP2 and 9 and extracellular matrix components like fibronectin and collagen [147]. Jong et al. identified lysyl oxidase-like 2 (LOXL2) on the surface of EVs released by human microvascular endothelial cells [148]. LOXL2 is involved in the remodeling of extracellular matrix proteins like fibronectin and collagen, promoting their cross-linking and contributing to the formation of the pre-metastatic niche [149].

**Fig. 2:**
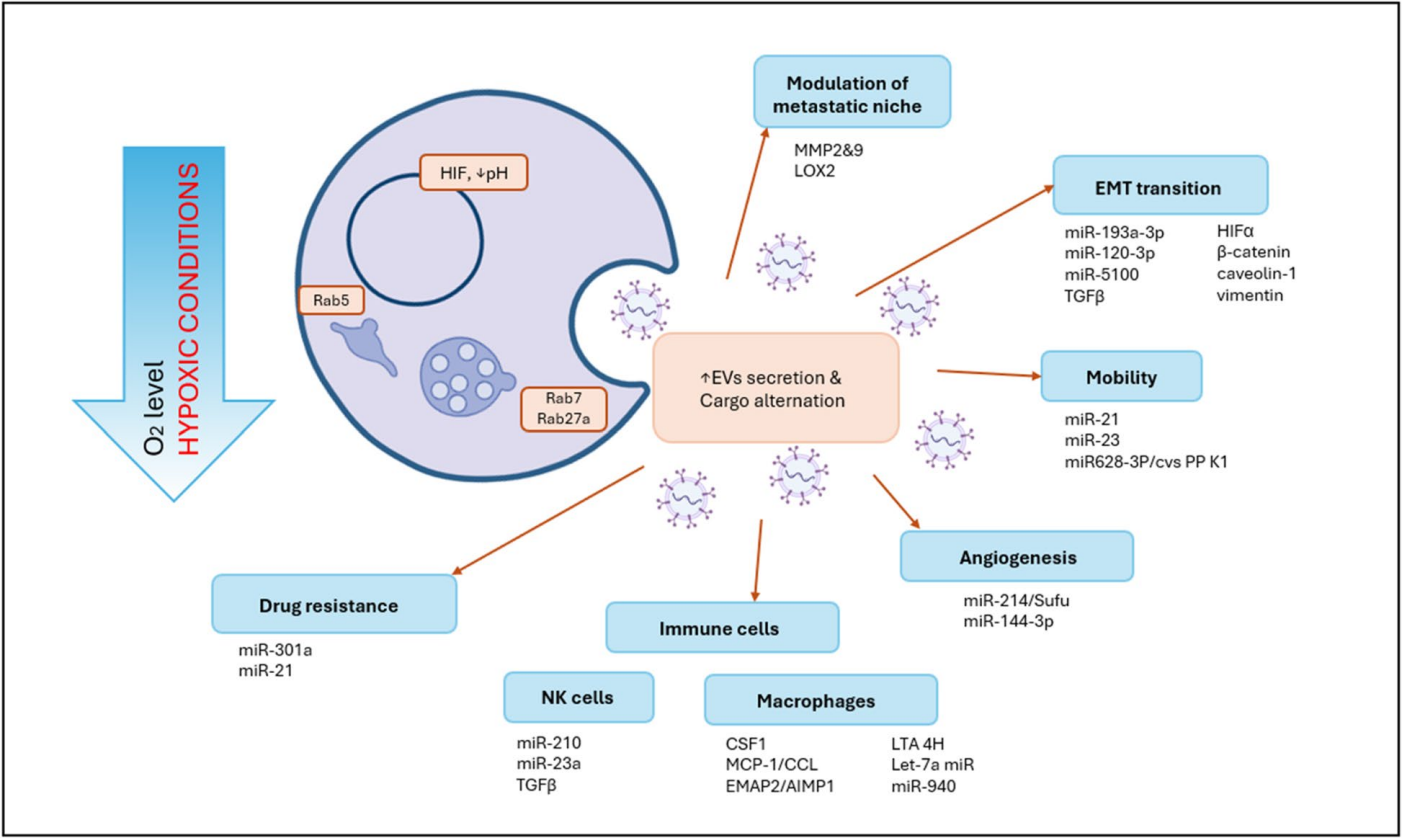
Hypoxia-induced adaptations in extracellular vesicle (EV) biogenesis and function in cancer progression. Activation of STAT3 under hypoxic conditions regulates Rab7 and Rab27a proteins to stimulate the production of EVs. Similarly, Rab5 regulates clathrin-coated vesicle-mediated transport from cell membrane to early endosomes and homotypic early endosome fusion, indicating a potential mechanism of early endosome formation and, consequently, the regulation of EV release. These hypoxia-adapted EVs modulate various aspects of cancer progression: metastatic niche preparation, epithelial-mesenchymal transition (EMT), enhanced cellular mobility, angiogenesis, drug resistance, and immunomodulation of immune cells. The diverse EV cargo, including specific miRNAs, proteins, and metabolites, orchestrates these multifaceted effects, illustrating the pivotal role of EVs in hypoxia-driven tumor adaptation and progression. Created in BioRender. Čapková, M. (2025) https://BioRender.com/s07i322

Furthermore, EVs secreted by hypoxic cells have been shown to increase the permeability of blood vessels, allowing cancer cells to enter the bloodstream and spread to distant organs. Li et al. reported that EVs released by oral squamous cell carcinoma cells under hypoxic conditions contain high levels of miR-21, which can induce growth and metastasis [150]. EVs derived from hypoxic lung adenocarcinoma cells have been shown to increase the transmigration of cancer cells by delivering miR-23, which regulates the tight junction protein ZO-1 in the endothelium [151]. Lin et al. demonstrated that a circRNA, circPDK1, induced by hypoxia, promotes pancreatic cancer cell proliferation, migration, and glycolysis. CircPDK1 may be activated mechanistically by HIF1α at the transcriptional level and by miR-628-3p, to activate the BPTF/c-myc axis [152]. All those secondary signs of experiencing hypoxia can be found on tumor-related EVs and contribute to the overall picture of the cancer progression and initiation of metastasis followed by the cancer spread. All those biomarkers can bring valuable information about ongoing changes in solid tumors and can point to a tendency to metastasize. More information on how hypoxia influences the EVs cargo and, therefore, contributes to cancer development is depicted in Fig. 2.

Integrins, a family of cell surface receptors involved in cell–matrix and cell–cell adhesion, are also very promising candidates for biomarkers [153, 154]. They bring valuable information for monitoring and/or predicting the metastatic spread of cancer. Moreover, they can help to predict the organotropism of the metastases. Hoshino et al. demonstrated that integrins on the surface of tumor-secreted EVs can direct organ-specific colonization by fusing with target cells in a tissue-specific manner, initiating the formation of the pre-metastatic niche [105]. These findings suggest that integrins not only facilitate adhesion but also activate signaling pathways and inflammatory responses in target cells, priming the organ for metastatic growth. Given the importance of integrins in organotropism and metastasis development, targeting integrins has emerged as a potential therapeutic strategy. Hoshino et al. demonstrated that integrin-blocking decoy peptides can successfully inhibit tumor EV adhesion in an integrin-specific and organ-specific manner [105]. Targeting specific integrins, such as αv, has shown promise in preventing metastasis to certain organs [105, 155–159]. Additionally, inhibiting α5β1, an upstream regulator of c-Met, Src, and FAK, has been shown to decelerate liver metastasis in mouse models of ovarian cancer and colorectal cancer [159–161].

There were several regulating RNAs identified to be connected with influence on metastasis (see Supplementary Table 4) The whole spectrum of RNAs delivered by EVs as its cargo can serve as prognostic markers. Moreover, those with suppressing influence in the metastatic spread hold significant promising potential in cancer treatment.

### EVs: important players in immune system surveillance and drug resistance

Immunity serves as a primary defense against cancer, and one of the critical cancer-eliminating mechanisms is mediated by the complement system. Cancer cells that fail to evade complement-mediated killing are typically eliminated at an early stage. However, for example, prostate cancer cells use EVs to disrupt the complement cascade through at least two key strategies [162]. First, they exhibit high activity of protein kinases A and C, as well as casein kinase II, which together phosphorylate the C3 complement component and thus inhibit its activation [163]. Second, these prostate cancer cell-derived EVs overexpress CD59, a well-known inhibitor of the membrane attack complex, blocking the final step of complement-mediated cell lysis [164].

**Fig. 3:**
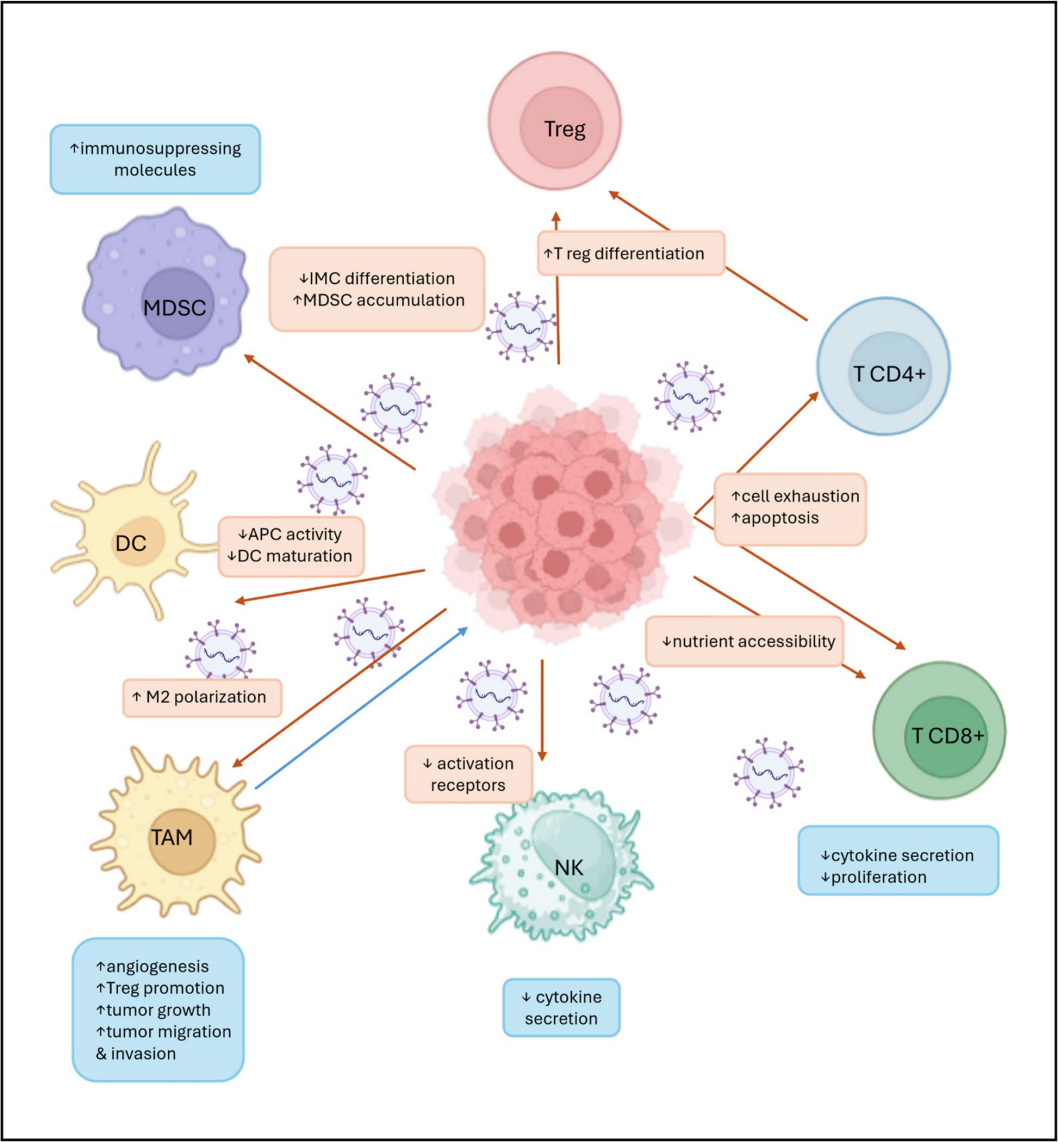
Immunomodulatory effects of tumor-derived extracellular vesicles (EVs) on the immune microenvironment. Schematic illustration of diverse impacts of tumor-derived EVs on various immune cell populations within the tumor microenvironment. EVs mediate multiple immunosuppressive mechanisms that collectively contribute to tumor immune evasion, promoting angiogenesis, tumor growth, and metastasis, thereby highlighting the crucial role of EVs in shaping the immunosuppressive tumor microenvironment. Created in BioRender. Čapková, M. (2025) https://BioRender.com/q08q189

Initiation of immune surveillance is a very dangerous situation. Tumor cells compromise the immune system’s vigilance and cause its failure in elimination of the tumor cells. Moreover, those tumors usually do not respond to immunotherapy. Aberrant expression of immune checkpoints (ICPs) usually plays a significant role, while cancer cells hijack the immunosuppressive effects of immunosuppressive ICPs to promote tumor progression. Several studies revealed that EVs-related ICPs have immunomodulatory effects and are involved in tumor immunity [165]. However, this is not the only strategy. Cancer cells employ various strategies to achieve this, including the secretion of large numbers of EVs that disrupt immune cell function and activate immunosuppressive cells. This contributes to the formation of a tumor-permissive microenvironment and to the tumor escape mechanism (see Fig. 3) [166].

#### Natural killers

Natural killer (NK) cells are crucial components of the innate immune system, capable of recognizing and eliminating tumor cells without prior activation [167]. However, tumor-derived EVs can effectively disarm NK cells, compromising their cytotoxic function and enabling tumor escape. One mechanism involves the downregulation of NKG2D, a key activating receptor on NK cells [168, 169]. Additionally, tumor-derived EVs can carry high levels of TNF-β, which can also reduce the expression of activating receptors NKp30 and NKG2D on NK cells [170–173].

Furthermore, tumor-derived EVs can interfere with NK cell cytokine production. While short-term exposure to these EVs may initially stimulate NK cell cytotoxicity, long-term exposure can inhibit their cytotoxic function [174]. EVs derived from various cancer types have been shown to significantly reduce the secretion of TNF-α and IFNγ by NK cells [168, 175], hindering their ability to orchestrate an effective anti-tumor immune response.

#### T-lymphocytes

Tumor-derived EVs exert a multifaceted suppressive effect on T-cells, orchestrating their dysfunction and ultimately facilitating immune evasion. One of the key strategies employed is metabolic reprogramming within the TME depleting the nutrients and starving the T-cells [176–181]. This nutrient depletion significantly impairs T-cell proliferation, cytokine secretion, and cytotoxic activity, hindering their ability to mount an effective anti-tumor response [182].

Beyond metabolic reprogramming, tumor-derived EVs can affect T-cells through various mechanisms, including the delivery of DNA, miRNAs, and suppressive protein ligands [183–188]. These interactions can inhibit T-cell proliferation, promote the expansion of regulatory T cells (Tregs), or trigger T-cell apoptosis and exhaustion.

Tumor-derived EVs can suppress T cell proliferation via transforming growth factor-ß [145] or hsa-miR-24-3p, hsa-miR-891a, hsa-miR-106a-5p, hsa-miR-20a-5p, and hsa-miR-1908 clusters, which down-regulate the MARK1 signaling pathway and alter cell proliferation and differentiation [186]. Additionally, EVs from mesothelioma cells have been shown to impair proliferative responses to IL-2 in CD4 + and CD8 + T cells [189]. EVs from nasopharyngeal carcinoma have been reported to impede the differentiation of immune-active Th1 and Th17 lymphocytes and induce the differentiation of immunosuppressive Tregs [186]. Specific miRNAs within EVs can also influence T-cell differentiation, such as miR-24-3p, which can inhibit the differentiation of Th1 and Th17 cells via repression of FGF11 [184], and miR-29a-3p and miR-21-5p, which can induce the Treg/Th17 cell imbalance [185].

T-cell exhaustion is another mechanism by which tumor-derived EVs can suppress anti-tumor immunity. Exhausted T cells exhibit reduced cytokine secretion, increased expression of inhibitory molecules, and a decreased ability to control tumor growth [190–192]. Tumor-derived EVs can induce T-cell exhaustion indirectly by activating anti-inflammatory M2 macrophages with EVs containing miR-146-5p or by delivering specific RNA cargo such as miR14-3-3ζ, circRNA-002178 [193–195].

Furthermore, tumor-derived EVs can induce T-cell apoptosis through mechanisms such as the activation of Fas ligand [188, 196]. EVs from pancreatic cancer cells can activate p38 MAP kinase signaling in T cells, leading to stress-mediated apoptosis [135].

#### Myeloid-derived suppressor cells

Myeloid-derived suppressor cells (MDSCs) play a significant role in immunosuppression and represent a challenge for many cancer immunotherapies [197].

Within the TME, cytokines produced by tumor cells, stromal cells, and activated immune cells induce the activation, expansion, and immunosuppressive activity of MDSCs [198–200]. Tumor-derived EVs contribute to MDSC survival by enhancing the expression of the anti-apoptotic protein Bcl-xL and activating the STAT1/3 pathway [201]. Additionally, these EVs can boost the production of suppressive molecules by MDSCs and enhance their suppressive activity in tumor models [107]. Furthermore, melanoma-derived EVs have been shown to promote the differentiation of myeloid cells into TGF-β-secreting cells while inhibiting their differentiation into dendritic cells (DCs) [202].

#### Neutrophils

Neutrophils, the most abundant type of white blood cell, play critical roles in tumor development. Within TME, cancer cells can regulate the behavior of neutrophils, transforming some into a pro-tumor phenotype [203].

Zhang et al. analyzed proteins derived from gastric cancer cells and found that EVs released by these cells contain high levels of high mobility group box 1 (HMGB1) [204]. HMGB1 plays a crucial role in initiating neutrophil pro-tumor activation, interacting with TLR4 to activate the TNF-β pathway and induce autophagy and pro-tumor activation of neutrophils via HMGB1/TLR4/NF-κB signaling [204].

Tumor-derived EVs can accumulate in lymphatic endothelium, creating a local chemotactic gradient involving CXCL8 that promotes neutrophil influx and the deposition of neutrophil extracellular traps (NETs). This microenvironment is favorable for tumor growth, suggesting that targeting NETs could be a potential therapeutic strategy [205].

#### Dendritic cells

DCs are crucial antigen-presenting cells that play a pivotal role in the immune response. However, in the context of cancer, DCs can be impaired, contributing to tumor development. DCs capture, process, and present tumor-derived antigens to T cells, but defects in DCs function can lead to antigen-specific tolerance [206–208].

Tumor-derived EVs play a significant role in inhibiting DCs function [209]. HLA-G, a non-classical MHC-I molecule expressed on tumor-derived EVs, can suppress T cells, NK cells, and DCs [210, 211]. Additionally, these EVs can inhibit the differentiation of DCs from bone marrow progenitors and monocytes, via prostanoids (e.g., PGE2) derived from cyclooxygenase-2, promoting the development of MDSCs [107, 202, 212–216].

Tumor-derived EVs can also impair DC maturation and antigen presentation [217]. Galectin-9 on the surface of glioblastoma multiforme-derived EVs can interact with the TIM3 receptor on DCs, inhibiting their antigen recognition, processing, and presentation [218, 219]. EVs enriched in S100A8 and S100A9 can also compromise DC maturation [220].

Furthermore, CD47, a protein expressed on tumor-derived EVs, can protect these EVs from phagocytosis by monocytes and macrophages [221]. This may allow EVs to avoid being taken up by DCs while still delivering their pro-tumorigenic contents. CD47 on EVs can also facilitate MDSC chemotaxis and migration, further impairing DCs maturation [197, 222].

#### Macrophages

Macrophages are highly plastic immune cells that can play both beneficial and harmful roles in cancer. While M1 macrophages have anti-tumor properties, M2 macrophages promote tumor growth, angiogenesis, and immunosuppression [223–225]. Tumor-derived EVs can actively shift the balance towards the pro-tumorigenic M2 phenotype, creating a tumor-favorable microenvironment [226].

Li et al. identified HMGB1 in EVs derived from esophageal squamous cell carcinoma and confirmed that EVs-related HMGB1 can trigger the differentiation of monocytes into PD1-positive tumor-associated macrophages, contributing to tumor progression [227].

One well-established mechanism by which tumor-derived EVs can reprogram macrophage function involves the transfer of miRNAs. For example, EVs containing miR-222 can target phosphatases and the PTEN gene, activating the Akt pathway and promoting M2 macrophage polarization [228, 229]. Similarly, upregulation of specific circRNAs such as hsa-circ0048117 or circFARSA can also promote M2 polarization in various cancer types [230]. These findings demonstrate the ability of tumor-derived cells to reprogram macrophage function by delivering the specific miRNA cargo.

Immunity functions as the primary barrier against cancer initiation and acts as the initial "treatment" to eradicate cancer cells. When this first line of defense fails (with the contribution of EVs), there are still several post-surgical strategies on how to minimize the cancer's progress and spread. Unfortunately, as with everything, cancer treatment has its limitations and weak points as well. One of them is drug resistance development.

#### Drug resistance

Approximately 90% of cancer-related deaths are associated with drug resistance [231]. Early distinguishing of the potential to drug resistance to a certain treatment is of high importance, as it can result in an inefficient treatment and loss of precious time, and, moreover, it helps to minimize the side effects of the treatment. EVs bring a new approach in recognition of drug resistance via liquid biopsy and also exhibit important examples of where the inhibition of EVs’ biogenesis can take its part.

EVs can facilitate drug resistance through several mechanisms, including the regulation of drug resistance genes and the horizontal transfer of molecules that confer resistance. EVs can also act as vehicles to remove drugs from cells and serve as mediators of drug efflux, they can also hide potentially dangerous signaling molecules that would attract the immune system.

Studies have shown that EVs derived from mesenchymal stem cells (MSCs) can regulate drug resistance-related proteins like lung resistance protein (LRP) and multi resistance protein (MRP), influencing the efficacy of chemotherapy drugs such as 5-fluorouracil and cisplatin [232–234]. Additionally, EVs can transfer multidrug resistance between cancer cells, facilitating the modulation of P-glycoprotein expression and affecting the transportation of anticancer agents and immunosuppressants [235].

EVs can also mediate the intercellular transfer of biomolecules between drug-resistant and drug-sensitive cells, resulting in altered gene expression in the recipient cell. Under hypoxic conditions, EVs can release miRNAs like miR21 and miR-301a, which can decrease recipient cell sensitivity to cisplatin and/or promote radiation resistance [236, 237].

The release of mitochondrial DNA (mtDNA) into the cytoplasm can initiate a DAMP signaling, leading to apoptosis ultimately resulting in the initiation of intrinsic apoptosis [238]. Cancer cells have evolved mechanisms to package and release mtDNA via EVs, avoiding the activation of DNA damage pathways and immunological responses [239–241]. This may allow cancer cells to evade apoptosis and immune activation. Furthermore, Sansone et al. demonstrated that EVs can harbor the full mitochondrial genome and transfer it to cells with impaired metabolism, restoring metabolic activity. This horizontal transfer of mtDNA in cancer stem-like cells can lead to increased self-renewal potential and resistance to hormonal therapy [242]. For the overview of cargo contributing to the EVs mediated drug resistance, see Supplementary Table 5.

In conclusion, there are several strategies on how EVs contribute to cancer development and progression. On the other hand, this is a double-edged sword, because EVs allow us to read the information as well and keep us with the progression and react to it. A comprehensive understanding of the contribution of EVs and signal molecule messages to cancer development can allow us to shift the treatment from subsequent and belated reaction to prompt interference to the initiated process.

## Potential of EVs in cancer diagnostics and treatment; current advances and future perspectives

### EVs in cancer diagnostic

EVs offer a valuable source of information for monitoring disease progression, particularly cancer. They are found in all body fluids and provide a non-invasive way to study the originating cells, their oncogenic transformations, TME, and immune system homeostasis. As we have already explained, EVs can offer insights into metastatic processes, organotropism, and drug resistance [243–245].

Selecting the most suitable biofluid for EV analysis and defining a robust panel of biomarkers that reflect the tissue of origin is critical for accurate determination of the disease status. Because tumor-derived EVs can ‘leak’ into circulation, their detection is feasible in various biofluids. The choice of which biofluid to analyze often depends on the tissue or organ of interest. For example, urinary EVs capture molecular and physiological/pathological changes in the kidney, urothelial tract, and gonads [246]. Likewise, cerebrospinal fluid–derived EVs may assist in the early detection of brain cancers, while tear-derived EVs have been proposed as a promising source of diagnostic and prognostic biomarkers for metastatic breast cancer and potentially other malignancies [247, 248].

While plasma is a rich source of EVs, it also contains other substances that can interfere with EV analysis, such as cells, cell-free DNA, and lipoproteins. Plasma EVs primarily originate from platelets, red blood cells, and leukocytes [243]. Tumor-derived EVs represent a small minority in blood samples, and their isolation can be challenging due to the presence of lipoproteins, which are present in much higher concentrations [245, 249].

Different EV isolation methods can yield varying results due to their differing efficacy in separating various types of EVs and other molecular entities [250, 251]. The choice of analytical method and its sensitivity is crucial for the effectiveness of liquid biopsy. Recent advancements in equipment sensitivity and assays have significantly improved the sensitivity of many analytical methods, reaching picomolar or femtomolar levels.

For accurate EV-biomarker analysis, meticulous attention must be paid to preanalytical variables, including sample collection, volume, preservatives, processing, and storage temperature [244, 252]. Samples should be stored at -80 °C for long-term storage to maintain EV integrity [253].

Isolating the targeted population of EVs from blood samples is crucial for liquid biopsy. Preconcentration techniques can enhance the sensitivity of EV-based analysis. Bioaffinity-based selection and concentration of EVs decorated with relevant markers, often achieved using microfluidic devices or advanced technologies like nanoscale Fluorescence Analysis and Cytometric Sorting (nanoFACS), are promising approaches [254, 255].

A challenge in EV isolation is that various methods may yield disparate results, likely due to the differing efficacy with which they segregate the distinct subtypes of EVs and other molecular entities present in the sample [250, 251].

Despite challenges in isolating specific subpopulations, EVs show considerable promise as biomarkers for early tumor diagnosis, prognosis prediction as well as treatment response assessment.

Early screening and accurate diagnosis are critical for improving patient outcomes and reducing cancer mortality. For example, a high prevalence of KRAS mutations in circulating exosomal DNA is observed in early-stage pancreatic cancer [256]. Elevated levels of GPC1^+^-circulating EVs are also a promising indicator, being significantly higher in patients with pancreatic ductal carcinoma and colorectal cancer compared to healthy individuals, suggesting their potential for early detection of digestive system cancers [257, 258]. In lung cancer, detecting EVs-based EGFR T790M offers a promising clinical diagnostic tool in non-small cell lung cancer [259]. EVs from breast cancer patients exhibited elevated levels of PKG1, RALGAPA2, NFX1, and TJP2 in the cancer group [260]. A panel of seven EV protein markers—EGFR, HER2, CA125, FRα, CD24, EpCAM, and CD9 + CD63 distinguished early-stage ovarian cancers from healthy controls [261]. Another study employed three EV proteins (FGG, MUC16, and APOA4) to discriminate early-stage ovarian cancers from benign cystadenoma/healthy controls [262]. It was demonstrated that EV proteins CD99, NGFR, ENO-2, EZR, and UGT3A2 are highly specific diagnostic biomarkers for Ewing sarcoma, using patient plasma samples [263]. Furthermore, several EVs related miRNAs, including miR-21-5p, miR-4454, and miR-720/3007a, are elevated in the urine of bladder cancer patients and could serve as early diagnostic biomarkers for this disease [264, 265].

EVs biomarkers hold significant prognostic potential, reflecting changes in tumor biology and predicting cancer behavior and patient survival. They also offer a promising approach for assessing treatment response, particularly drug resistance, a major obstacle in advanced cancers. Several studies highlight the role of EVs in metastasis. Keklikoglou et al. showed that cytotoxic chemotherapy can promote breast cancer metastasis by inducing the secretion of annexin A6-enriched EVs [266]. These EVs are then transferred to lung endothelial cells, creating a pre-metastatic niche. Exosome-associated Annexin II and L-plastin also contribute to metastasis and may serve as prognostic markers in advanced breast cancer [131, 267]. In colorectal cancer, overexpression of miR-193a in EVs is a potential biomarker for liver metastasis. Zeng et al. demonstrated that tumor-derived exosomal miR-25-3p promotes colorectal cancer to liver metastasis by increasing vascular permeability and angiogenesis [268]. A clinical trial in rectal cancer found elevated plasma exosomal miR-141-3p and miR-375 in patients with liver metastasis [269]. Similarly, miR-21, miR-18a, miR-17-5p, and miR‐548c‐5p may serve as early screening markers for colorectal to liver metastasis [270–273]. In prostate cancer, urinary exosomal ITGA3 and ITGB1 are upregulated in metastatic patients compared to those with benign tumors and early-stage cancer [274].

EVs also show promise in predicting recurrence and survival. In triple-negative breast cancer patients with residual disease after neoadjuvant therapy, a plasma EV miRNA profile (miR-200a-3p, miR-203a-3p, and miR-7845-5p) correlated with increased recurrence risk. This profile could help identify high-risk patients and guide adjuvant treatment decisions [275].

Furthermore, EVs can predict treatment response and resistance. In neuroblastoma, an EV microRNA signature (miR-29c, miR-342-3p, and let-7b) predicts clinical responders [276]. Elevated miR-425-3p, on the other hand, predicts poor response to cisplatin in non-small cell lung carcinoma [277]. Gastrointestinal stromal tumor-derived EVs carry proteins that can track disease burden and predict response to targeted therapy [278]. Finally, several studies have shown that PD-L1 on EVs contributes to tumor immune evasion and can predict response to therapy and adaptive resistance [279, 280]. Porcelli et al. found that uPAR-positive EVs in metastatic melanoma patients are associated with resistance to checkpoint inhibitor immunotherapy [281].

Overall, plasma and urine remain the two most commonly examined body fluids for EV-based diagnostics; plasma as a circulating biomarker reservoir and urine as a direct route for tumors arising in the genitourinary tract. Despite technological and methodological hurdles, continuous advances in bioaffinity-based selection, microfluidic devices, and high-sensitivity assays promise to overcome these challenges, broadening the clinical application of EV-based liquid biopsy.

### EVs in cancer therapy

EVs can also exhibit great potential as targeted drug-delivery nanocarriers (DDN). The rise of EV-based therapies is gaining momentum due to the safer profile and easier manufacturing, storage, and clinical use of EVs compared to cell-based therapies. However, challenges related to purity, identity, and safety must be addressed [282–285]. Several guidelines and recommendations have been published to facilitate the introduction of EVs in clinical trials, including requirements for EV isolation, characterization, and potency tests [7–9, 286–288]. The urge for unified procedures for EV implementation into medicinal practice can be illustrated by the number of clinical trials employing EVs as biomarker source or therapeutic agens (see Supplementary Table 6).

#### EVs as drug delivery nanocarriers

**Fig. 4:**
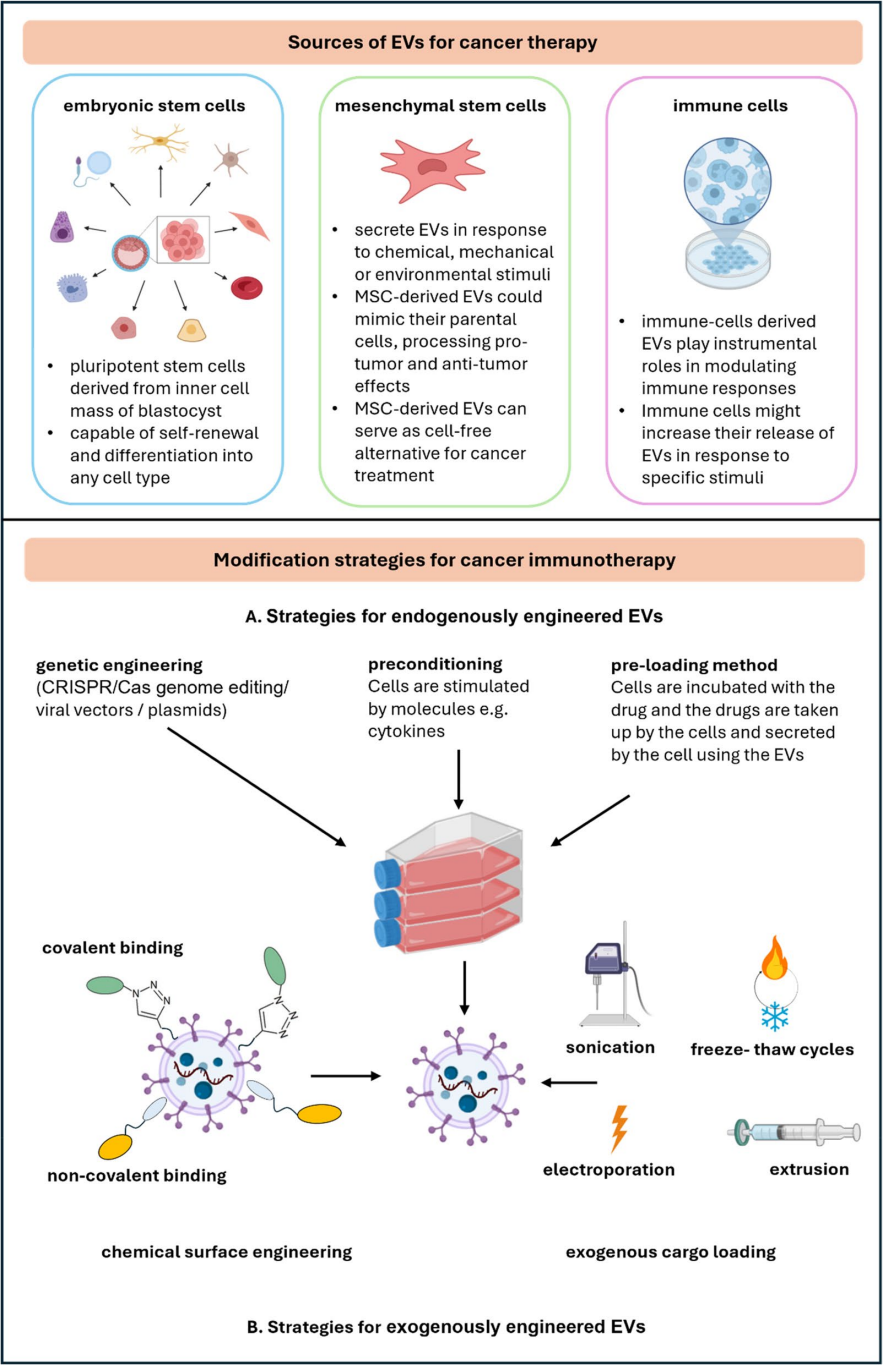
Modification strategies of extracellular vesicles (EVs) for cancer immunotherapy. Current strategies for EVs engineering are generally divided into two categories: A. endogenous engineering including gene engineering and cell-related processes to insert the cargo of interest; and B. exogenous engineering utilizing chemical methods for surface modification to display of ligands or receptors on the EVs surface or physical methods, such as electroporation, sonication, freeze–thaw cycles, and extrusion, to increase permeability of the EVs membrane and to facilitate the loading of the cargo of interest. Created in BioRender. Čapková, M. (2025) https://BioRender.com/o12r208

An increasing number of studies in recent years have explored the use of EVs as DDNs because of their advantageous features, including low immunogenicity and high biocompatibility. MSCs and immune cells are among the principal in vitro sources of EVs intended for drug delivery (see Fig. 4). For safety reasons, using EVs derived directly from tumor cells is generally avoided, as cancer EVs could inadvertently promote tumor invasion or epithelial-mesenchymal transition, or even transfer tumor resistance genes [282, 284].

A comparative evaluation of EVs derived from different cell lines and their respective biodistribution patterns revealed that although EVs primarily accumulate in the liver, lung, spleen, and gastrointestinal tract, the originating cell type and route of administration markedly affect biodistribution. For example, EVs derived from dendritic cells tend to localize preferentially to the spleen, whereas melanoma cell-derived EVs predominantly accumulate in the liver [289]. Systemic EVs administration often leads to non-specific accumulation in the liver, spleen, gastrointestinal tract, and lung, yet native EVs can also show notable accumulation in tumor tissue [289, 290].

Despite these targeting advantages, the terminal half-life of EVs remains relatively short. Even when stealth properties have been implemented (e.g., polyethylene glycol modification), the terminal half-life of EVs has reached at most about 60 min [291]. Although EVs have long been considered biocompatible due to their mammalian origin and “physiological” composition, such broad generalizations are not advisable. Indeed, while transfusion of blood-cell-derived EVs (e.g., platelet-derived EVs) usually does not provoke major adverse effects, there have been occasional associations with transfusion-related acute lung injury [292], emphasizing that the immunogenicity and biocompatibility of each individual EV formulation must be rigorously evaluated—much like any drug delivery nanocarrier.

To date, most approaches have focused on producing EVs from healthy human cell lines to minimize immunogenicity. In one example, intravenous and intraperitoneal administration of EVs derived from human embryonic kidney cells to mice for three weeks showed no observable toxic effects [293]. Data from non-human primate studies are similarly encouraging. Nonetheless, every EV-based carrier must undergo specific safety and immunogenicity assessments. Although autologous EVs have been proposed, collecting and culturing patient’s own cells to produce vesicles for re-administration, most applications favor well-characterized, non-autologous EV sources [294]. This preference stems from practical considerations such as scalability, regulatory constraints, and a desire for standardized, rigorously qualified products. Of note, non-engineered, non-autologous EVs have already been administered to human subjects in numerous clinical studies with good safety outcomes [283].

Currently, MSC-derived EVs are being tested for regenerative medicine, and dendritic-cell-derived EVs are in development for vaccine delivery. Both have demonstrated encouraging safety profiles in several phase I clinical trials [295]. Although additional refinements in EV engineering and manufacturing are necessary to optimize targeting, half-life, and release kinetics, these ongoing clinical investigations underscore the feasibility of leveraging EVs as next-generation, precision drug carriers in cancer therapy [296, 297].

#### Engineered EVs as drug delivery nanocarriers

Technological advancements help to overcome limited clinical application of natural EVs such as low drug delivery efficacy and insufficient antitumor capacity. Engineered EVs might be loaded with different therapeutic cargo, and preferentially target tumor sites and therefore exert great potential for cancer therapy and immunotherapy [298]. Avenues for EVs modifications and EVs-based therapeutic strategies have been already reviewed [299–304].

There are basically two kinds of approaches for EVs modification and utilization in EVs-based therapies: (i) endogenous modification: the biogenesis machinery is used for incorporation of the cargo (the parental cells are often genetically altered or stimulated before EVs isolation), (ii) exogenous modification: drugs and therapeutic agents are directly encapsulated into EVs or the EVs surface is decorated after their secretion out of the parental cells.

##### Strategies for endogenously engineered EVs

CRISPR/Cas genome editing has revolutionized cell engineering, enabling precise modifications of cellular DNA. EVs were utilized for targeted delivery of the CRISPR/Cas9 components, including plasmids [305, 306], mRNA [307, 308], and even the pre-assembled ribonucleoprotein complex to edit the cancer cells. However, developing safe and effective in vivo delivery systems remains the key obstacle to realizing the full potential of CRISPR-Cas9 gene therapies [309, 310]. To meet the safety criteria CRISPR/Cas9 can be utilized to engineer the cells in vitro. By incorporating genes of interest into parental cells, it's possible to engineer these cells to express desired membrane-bound proteins. Those endogenously engineered EVs can be utilized, for example, in restarting the immune system vigilance and simulation of natural processes leading to cancer clearance. These overexpressed proteins can then be transferred to EVs, modifying their surface composition [311–314].

Membrane-tethering technology for proteins is a promising approach for developing therapeutic agents. This technique involves fusing bioactive proteins, such as cytokines, with membrane-targeting sequences, allowing them to be displayed on the cell surface and induce autocrine signaling [315]. Conditioning cells with added free cytokines, especially immune cells, can also influence the properties of EVs they produce. For example, conditioning DCs or macrophages with IFN-γ can enhance the anti-tumor efficacy of their EVs [316, 317]. Stimulating NK cells with IL-15 and IL-21 can enhance their cytotoxic activity against cervical or lung cancer cells [318]. Additionally, EVs can be engineered to carry specific miRNAs or small interfering RNA (siRNA) molecules to promote or suppress the production of certain genes [319, 320].

As it was described previously, CD47 expressed on tumor-derived EVs can protect these EVs from phagocytosis by monocytes and macrophages. This “don't eat me” signal can be used to improve the retention time of engineered EVs in the bloodstream, because it enables EVs to escape from clearance by the mononuclear phagocytic system [321].

Exosomes derived from tumor cells hold promise as cancer vaccines. These EVs can present tumor antigens to immune cells, triggering an anti-tumor immune response [322, 323]. DC-derived EVs, which express MHC-I and MHC-II molecules, can induce regression of tumors through cytotoxic T lymphocyte (CTL) activation [324, 325]. Conditioning DCs with tumor-specific antigens can enhance CTL responses, and LPS-stimulated DC-EVs have shown strong T-cell activation [326, 327].

##### Strategies for exogenously engineered EVs

Exogenously engineered EVs are the sort of exosomes modified with surface decoration and internal therapeutic molecules. After appropriate modification, engineered EVs are able to deliver antitumor drugs to tumor sites effectively and help to decrease the side effects of the treatment (see Fig. 4). There are several benefits to enhancing the therapeutic effect of EVs: (i) improved pharmacokinetics, (ii) improved targeting of the tumor sites, and (iii) improved drug release.

In exogenous engineering, drugs and therapeutic agents are directly encapsulated into EVs [328, 329]. This can be achieved via two different mechanisms: the diffusion of therapeutic agents into the lumen of EVs along a concentration gradient or the formation of transient pores in the EVs' membrane to allow the cargo to cross. This approach allows direct control over the inserted cargo composition and offers a more reproducible mechanism to control the concentration. Therapeutic agents can be represented by specific nucleic acids, proteins, cytostatics, or agents for photothermal/photodynamic therapy and maybe in the future also gene therapy that can interfere with tumor progression [330, 331].

Surface modification of EVs can enhance their ability to target specific tissues or cells. By mimicking strategies employed by cancer cells themselves, EVs can be designed to home to metastatic sites. For example, EVs associated with chemokines like CXCR4 can be beneficial for targeted delivery [332, 333]. CXCR4 exhibits a special affinity for SDF-1, a factor widely expressed on tumor surfaces. This interaction can facilitate the aggregation of MSCs at the tumor site. However, EVs expressing high levels of CXCR4 can be used as vehicles for the precise delivery of therapeutic agents. Xu et al. demonstrated that EVs loaded with siRNA can effectively accumulate at tumor sites and suppress the Survivin gene, inhibiting tumor growth [334].

Surface modification by antibodies can also take advantage of classical cancer treatment targets, for example, targeting HER-2-positive cancer using antibodies such as trastuzumab or tucatinib. Those antibodies can serve as components of the classical treatment, moreover, they can potentially also “bring” another portion of drugs or receptor silencing agents [335, 336].

Engineered EVs (CDK-004) were designed for the treatment of advanced hepatocellular carcinoma and liver metastases [337]. CDK-004 is designed to deliver the STAT6 antisense oligonucleotide to myeloid cells, repolarizing macrophages from an immunosuppressive M2 phenotype to a pro-inflammatory M1 phenotype, potentially leading to antitumor activity.

EVs loaded with conventional anti-cancer drugs can exhibit improved therapeutic efficacy compared to the drugs alone, often with reduced systemic toxicity. For example, EVs loaded with doxorubicin or paclitaxel can achieve pronounced antitumor effects while minimizing major organ damage [338, 339].

Further engineering of EVs can enhance their therapeutic potential. For instance, EVs loaded with doxorubicin and AgS_2_ quantum dots can be designed for controlled release under near-infrared) irradiation, enabling targeted drug delivery to tumors and improving the penetration depth of the drug [340]. Additionally, EVs can be engineered to carry other photothermal agents, such as indocyanine green, or photosensitizers and ferroptosis inducers, for use in photodynamic therapy [341–343].

Precision engineering of EVs is essential for their effective therapeutic application. By modifying the surface of EVs to target specific tumor sites and optimizing their pharmacokinetics, EVs can serve as cargo vehicles for delivering therapeutic agents. This approach holds promise for improving the efficacy and safety of cancer treatments.

### Cancer therapy-mediated changes on EVs cargo

Cancer therapies like radiotherapy and chemotherapy induce cellular stress through various mechanisms, including reactive oxygen species (ROS) production, DNA damage, and organelle damage, often triggering autophagy [344–346]. These stresses significantly alter the cargo of released EVs, a complex process influenced by cancer type and specific treatment regimens [347]. Cancer cells frequently increase EV production and release, potentially as a survival mechanism to discard damaged components or signal stress to neighboring cells [348].

ROS, crucial in cell proliferation, motility, the cell cycle, and apoptosis [349, 350], can be highly toxic to both tumor and normal cells [351]. TNF-α amplifies this toxicity by boosting ROS production and mitochondrial dysfunction [352–354]. Oxidative stress can impact EV biogenesis, and EVs themselves may reflect therapy-induced oxidative damage, potentially contributing to cognitive impairment in some cancer survivors. For example, the reaction between 4-hydroxy-2-nonenal and proteins, a hallmark of oxidative stress, can lead to protein misfolding and proteasome dysfunction [344, 355]. EVs may then serve as a pathway for eliminating these oxidized proteins, as demonstrated by increased EV generation following doxorubicin treatment (an oxidative stress inducer) [356]. However, the precise impact of oxidative stress on EV biogenesis requires further study.

DNA damage, another consequence of cancer therapy, can lead to the release of DNA into circulation via EVs [357, 358]. Increased DNA packaging in EVs is observed in genotoxic conditions and cancer [359]. Genotoxic drugs elevate micronuclei production and exosome release [360]. Micronuclei and exosomes can interact, sharing nuclear proteins, with CD63 facilitating nuclear material transfer into exosomes. This raises the question of whether these DNA-carrying EVs can be internalized by recipient cells and how this affects the recipient cell population.

Studies have also shown that DNA within apoptotic bodies can mediate horizontal gene transfer [361]. This has also been observed with EVs carrying DNA, which can integrate into recipient cell genomes [362]. EV-DNA can even increase the expression of corresponding mRNA and proteins [363], as demonstrated with the transfer of the BCR/ABL fusion gene in chronic myeloid leukemia [364]. Oncogenic H-ras fragments can also be transferred via EVs, increasing proliferation in recipient cells [365], although these changes may not always be permanent [366]. Furthermore, mtDNA transfer via EVs has been linked to therapy resistance, for example in breast cancer [242].

Finally, therapeutic stress can induce endoplasmatic reticulum (ER) stress and trigger the unfolded protein response (UPR) [367–369]. The UPR, linked to exosome secretion and autophagy, can be modulated by ER stress induced by chemotherapy and radiotherapy. This interplay between therapy-induced UPR, autophagy, and EV secretion represents a critical adaptive mechanism that may influence cancer cell survival and treatment outcomes.

EVs unique cargo composition mirrors ongoing tumor processes and provides a minimally invasive means to gauge disease progression, predict treatment responses, and identify emerging drug resistance. Moreover, EV-based strategies show potential for precision drug delivery and immunotherapy, as they can be engineered to enhance targeted treatment efficacy while minimizing adverse effects. Continued research into EV biology and refinement of isolation and characterization techniques will be crucial to harness their full clinical potential, ultimately paving the way toward more personalized and effective cancer care.

## Conclusion

EVs have emerged as crucial mediators of intercellular communication in cancer, orchestrating a complex network of interactions that influence tumor progression, metastasis, immune evasion, and therapeutic response. Their diverse cargo, mirroring the dynamic cellular landscape, provides a rich source of information for understanding the intricate mechanisms driving cancer development and holds immense potential for revolutionizing cancer diagnostics and treatment.

The potential of EVs as diagnostic and therapeutic tools is rapidly expanding. Liquid biopsies utilizing EVs offer a minimally invasive approach for monitoring cancer progression and treatment response in real time. By analyzing the cargo of EVs isolated from body fluids, we can gain valuable information about tumor origin, metastatic potential, and emerging drug resistance, enabling earlier interventions and personalized treatment strategies.

As we continue to unravel the intricate mechanisms governing EVs production and function, we can anticipate further advancements in utilizing EVs for both diagnostic and therapeutic applications.

In conclusion, EVs represent a powerful tool for navigating the complex landscape of cancer. By deciphering the messages carried by these vesicles, we can gain a deeper understanding of cancer biology and develop more effective strategies for early detection, targeted therapy, and immunotherapy. On one side, EVs contribute to cancer development; on the other side, they are offering a new era of personalized and precise medicine with the potential to significantly improve patient outcomes.

## Supplementary Information

Supplementary Material 1.

Supplementary Material 2.

Supplementary Material 3.

Supplementary Material 4.

Supplementary Material 5.

Supplementary Material 6.

## Data Availability

No datasets were generated or analysed during the current study.

## Abbreviations

CAF: cancer-associated fibroblast
CAP: cancer-associated protein
circRNA: circular RNA
CTL: cytotoxic T lymphocyte
DAMP: damage-associated molecular pattern
DC: dendritic cell
DDN: drug delivery nanocarriers
EMT: epithelial-mesenchymal transition
ER: endoplasmatic reticulum
ESCRT: endosomal sorting complex required for transport
EV: extracellular vesicle
Gal-3: galectin 3
GPR143: G-protein coupled receptor 143
HMGB1: high mobility group box 1
Hsp90: heat shock protein 90
ICP: immune check point
ILV: intraluminal vesicle
lncRNA: long non-coding RNA
LOXL2: lysyl oxidase-like 2
LRP: lung resistance protein
MDSC: myeloid-derived suppressor cell
miRNA: micro RNA
MMP: matrix metalloproteinase
MP: membrane protein
mRNA: messenger RNA
MRP: multi resistance protein
MSC: mesenchymal stem cell
mtDNA: mitochondrial DNA
MVB: multivesicular body
nanoFACS: nanoscale Fluorescence Analysis and Cytometric Sorting
NET: neutrophil extracellular trap
NK: natural killer
piRNA: piwi-interacting RNA
ROS: reactive oxygen species
rRNA: ribosomal RNA
siRNA: small interfering RNA
snoRNA: small nucleolar RNA
TME: tumor microenvironment
Tregs: regulatory T cells
tRNA: transfer RNA
UPR: unfolded protein response
VEGF: vascular endothelial growth factor
vtRNA: vault RNA
