# Supplementary material for: Extracellular vesicles in cancer´s communication: messages we can read and how to answer
Source: Mol Cancer. 2025 Mar 19;24:86. doi: 10.1186/s12943-025-02282-1 (PMC11921637; doi:10.1186/s12943-025-02282-1)
Supplement: Supplementary file 1 — Supplementary Material 1. [file 12943_2025_2282_MOESM1_ESM.docx]

**Table S1: EV-associated RNAs implying ongoing cancer-related changes**

| **Protumorigenic effect** | | | | | |
| --- | --- | --- | --- | --- | --- |
| *RNA* | *Tissue* | *Affected systems* | *Effect* | *In vitro/ in vivo* | *Ref.* |
| circR-000166 | Colorectal cancer | ↓ miR-326 ↑ LASP1 | ↑ apoptosis, ↑ cell growth | In vitro | [1] |
| circR-0008928 | Non-small cell lung cancer | ↓ miR-488 ↑ HK2 | ↑ glycolysis, ↑ cancer progression, ↓ sensitivity to cisplatin | In vitro | [2] |
| circR-102179 | Non-small cell lung cancer | ↓ miR-330-5p ↑ HMGB3 | ↑ invasion, ↑ migration, ↑ proliferation | In vitro, in vivo | [3] |
| circR-ABCC1 | Colorectal cancer | ↑ Wnt/β-catenin pathway | ↑ invasion, ↑ stemness | In vitro | [4] |
| circR-Cdr1as | Hepatocellular carcinoma | ↓ miR-1270 ↑ AFP | ↑ migration, ↑ proliferation | In vitro, in vivo | [5] |
| circR-PDE8A | Pancreatic cancer | ↓ miR-338 ↑ MACC1, MET | ↑ progression, ↑ proliferation | In vitro, ex vivo | [6] |
| circR-RANBP17 | Neuroblastoma | ↓ miR-27b-3p ↑ KDM1A | ↓ apoptosis, ↑ invasion, ↑ migration, ↑ proliferation | In vitro, in vivo | [7] |
| circR-WHSC1 | Ovarian cancer | ↓ miR-145, miR-1182 ↑ MUC1, hTERT | ↓ apoptosis, ↑ invasion, ↑ migration, ↑ proliferation | In vitro, in vivo | [8] |
| lncR-CAF | Oral squamous cell carcinoma | ↑ IL-33 | ↑ invasion, ↑ proliferation | In vitro, in vivo | [9] |
| lncR-CASC15 | Osteosarcoma | ↓ miR-338-3p ↑ RAB14 | ↑ invasion, ↑ migration, ↑ proliferation | In vitro, in vivo | [10] |

| **Protumorigenic effect** – continued | | | | | |
| --- | --- | --- | --- | --- | --- |
| *RNA* | *Tissue* | *Affected systems* | *Effect* | *In vitro/*  *in vivo* | *Ref.* |
| lncR-ENO1-IT1 | Colorectal cancer | ↑ SP1 | ↑ glycolysis | In vitro | [11] |
| lncR-GS1-600G8.5 | Breast cancer | ↓ tight junction proteins | ↑ passage across the blood-brain barrier | In vitro, in vivo | [12] |
| lncR-MALAT-1 | Non-small cell lung cancer | *n.s.* | ↓ apoptosis, ↑ migration, ↑ proliferation | In vitro | [13] |
| lncR-MYU | Prostate cancer | ↓ miR-184 ↑ c-Myc | ↑ migration, ↑ proliferation | In vitro | [14] |
| lncR-PCSEAT | Prostate cancer | ↓ miR-143-3p, miR-24-2-5p ↑ EZH2 | ↑ growth, ↑ motility | In vitro | [15] |
| lncR-SBF2-AS1 | Pancreatic cancer | ↓ miR-122-5p ↑ XIAP | ↑ progression | In vitro, in vivo | [16] |
| miR-100-5p, miR-139-5p, miR-21-5p | Prostate cancer | ↑ MMP-2, -9, -13, RANKL | ↑ metastasis, ↑ osteoblast differentiation, ↑ progression | In vitro | [17] |
| miR-10b-5p | Gastric cancer | ↓ KLF11 ↑ TGFβR1 | ↑ migration, ↑ proliferation | In vitro | [18] |
| miR-1247-3p | Hepatocellular carcinoma | ↓ B4GALT3, ↑ β1-integrin–NF-κB signalling | ↑ fibroblast activation, ↑ lung metastasis, ↑ progression | In vitro | [19] |
| miR-146b-5p | Thyroid cancer | ↓ SMAD4, TGF-β pathway | ↑ proliferation | In vitro | [20] |
| miR-193b-3p | Pancreatic cancer | ↓ TRIM62 | ↑ glutamine uptake, ↑ invasion, ↑ migration, ↑ proliferation | In vitro, in vivo | [21] |
| miR-196a-5p | Thyroid cancer | ↓ LRP1B | ↑ invasion, ↑ migration, ↑ proliferation | In vitro | [22] |

*n.s.: not specified*

| **Protumorigenic effect** - continued | | | | | | |
| --- | --- | --- | --- | --- | --- | --- |
| *RNA* | *Tissue* | *Affected systems* | *Effect* | *In vitro/ in vivo* | *Ref.* |  |
| miR-19b-3p | Esophageal cancer | ↓ PTEN | ↓ apoptosis, ↑ invasion, ↑ migration | In vitro | [23] |  |
| miR-21 | Glioma | ↓ Btg2, Nfat5 and Pdcd4 mRNA | ↑ proliferation | In vitro, in vivo | [24] |  |
| miR-21a | Lung cancer | ↓ PDCD4 | ↓ apoptosis, ↑ migration, ↑ proliferation | In vitro, in vivo | [25] |  |
| miR-224-5p | Renal cancer | *n.s.* | ↑ invasion, ↑ proliferation | In vitro | [26] |  |
| miR-3648, miR-4258, miR-638, miR-663a | Hepatocellular carcinoma | ↓ VE-cadherin, ZO-1 | ↑ metastasis, ↑ vascular permeability | In vitro, in vivo | [27] |  |
| miR-410-3p | Colorectal cancer (hypoxic conditions) | ↓ PTEN ↑ PI3K/Akt signaling | ↑ invasion, ↑ migration, ↑ proliferation | In vitro, in vivo | [28] |  |
| miR-92a | Lung cancer | ↓ SMAD7 ↑ TGFβ signaling | ↑ formation of hepatic pre-metastatic niche | In vitro, in vivo | [29] |  |
| miR-93-5p | Esophageal cancer | ↓ PTEN, p21 ↑ cyclin D1 | ↑ proliferation | In vitro | [30] |  |

*n.s.: not specified*

| **Suppressive effect** | | | | | | | | | | |
| --- | --- | --- | --- | --- | --- | --- | --- | --- | --- | --- |
| *RNA* | | *Tissue* | | *Affected systems* | | *Effect* | | *In vitro/ in vivo* | | *Ref.* |
| circR-STAU2 | | Gastric cancer | | ↓ miR-589 ↑ CAPZA1 | | ↓ progression | | In vitro, in vivo | | [31] |
| circR-0051443 | | Hepatocellular carcinoma | | ↓ miR-331-3p ↑ BAK1 | | ↑ apoptosis, ↓ tumor growth | | In vitro, in vivo | | [32] |
| lncR-LINC01133 | | Bladder cancer | | ↓ Wnt pathway | | ↓ epithelial-mesenchymal transition ↓ tumor growth | | In vitro, in vivo | | [33] |
| lncR-PTENP1 | | Bladder cancer | | ↓ miR-17 ↑ PTEN | | ↑ apoptosis, ↓ invasion, ↓ migration ↓ tumor growth | | In vitro, in vivo | | [34] |
| miR-100, miR-125b | | Hepatocellular carcinoma | | ↓ IGF2, AKT/mTOR pathway | | ↓ tumor growth | | In vitro, in vivo | | [35] |
| miR-10a | | Colorectal cancer | | ↓ IL‑6, IL‑8, IL‑1β | | ↓ migration, ↓ proliferation | | In vitro | | [36] |
| miR-1228 | | Gastric cancer | | ↓ MMP-14 | | ↓ development, ↓ progression | | In vitro | | [37] |
| miR-126 | | Non-small cell lung cancer | | ↑ PTEN ↓ PI3K, p-AKT | | ↓ migration, ↓ proliferation | | In vitro, in vivo | | [38] |
| miR-127-3p | | Choriocarcinoma | | ↓ ITGA6 | | ↑ apoptosis, ↓ invasion, ↓ migration, ↓ proliferation | | In vitro | | [39] |
| miR-134-5p | | Breast cancer | | ↓ ARHGAP1, PI3K/AKT pathway | | ↑ apoptosis, ↓ invasion, ↓ migration, ↓ proliferation | | In vitro, in vivo | | [40] |
| miR-16-5p | | Lung cancer | | ↓ PD-L1 | | ↑ apoptosis, ↓ migration, ↓ proliferation | | In vitro, in vivo | | [41] |
| **Suppressive effect** - continued | | | | | | | | | | |
| *RNA* | *Tissue* | | *Affected systems* | | *Effect* | | *In vitro/ in vivo* | | *Ref.* | |
| miR-185-5p | Non-small cell lung cancer | | ↓ RAB35 | | ↓ invasion, ↓ migration, ↓ proliferation | | In vitro, in vivo | | [42] | |
| miR-22-3p | Colorectal cancer | | ↓ RAP2B, PI3K, p-AKT | | ↓ invasion, ↓ proliferation | | In vitro | | [43] | |
| miR-23b-3p | Cervical cancer | | ↓ c-Met | | ↓ invasion, ↓ migration, ↓ proliferation | | In vitro, ex vivo | | [44] | |
| miR-320a | Lung cancer | | ↓ SOX4 | | ↓ cancer cell growth | | In vitro, in vivo | | [45] | |
| miR-34a | Pancreatic cancer | | ↓ Bcl-2 | | ↓ cancer cell growth | | In vitro, in vivo | | [46] | |
|  | Breast cancer | | ↓ Bcl-2, c-Met | | ↑ apoptosis, ↓ invasion, ↓ migration | | In vitro | | [47] | |
| miR-375 | Esophageal cancer | | ↓ ENAH | | ↑ apoptosis, ↓ invasion, ↓ migration, ↓ proliferation | | In vitro, in vivo | | [48] | |
| miR-495, miR-5688 | Non-small cell lung cancer | | ↓ IL-11 | | ↓ invasion, ↓ migration, ↓ proliferation | | In vitro, in vivo | | [49] | |
| miR-503-3p | Endometrial cancer | | ↓ MEST | | ↓ progression | | In vitro, in vivo | | [50] | |
| miR-5100 | Breast cancer | | ↓ CXCL12/CXCR4 axis | | ↓ epithelial-mesenchymal transition, ↓ invasion, ↓ migration | | In vitro, in vivo | | [51] | |

| **Suppressive effect** - continued | | | | | |
| --- | --- | --- | --- | --- | --- |
| *RNA* | *Tissue* | *Affected systems* | *Effect* | *In vitro/ in vivo* | *Ref.* |
| miR-6127, miR-6746-5p, miR-6787-5p | Colorectal cancer | ↑ RAPA ↓ mTOR pathway | ↑ EV production,  regulation of pre-metastatic niche | In vitro | [52] |
| miR-99b, miR-203-3p | Gastric cancer | ↓ IGF-1R | ↓ tumor growth | In vitro | [53] |

1. Hao Q, Zhang Z. hsa_circRNA_000166 Facilitated Cell Growth and Limited Apoptosis through Targeting miR-326/LASP1 Axis in Colorectal Cancer. Gastroenterol Res Pract. 2020;2020:8834359. 10.1155/2020/8834359.

2. Shi Q, Ji T, Ma Z, Tan Q, Liang J. Serum Exosomes-Based Biomarker circ_0008928 Regulates Cisplatin Sensitivity, Tumor Progression, and Glycolysis Metabolism by miR-488/HK2 Axis in Cisplatin-Resistant Nonsmall Cell Lung Carcinoma. Cancer Biother Radiopharm. 2023;38(8):558-71. 10.1089/cbr.2020.4490.

3. Zhou ZF, Wei Z, Yao JC, Liu SY, Wang F, Wang Z, et al. CircRNA_102179 promotes the proliferation, migration and invasion in non-small cell lung cancer cells by regulating miR-330-5p/HMGB3 axis. Pathol Res Pract. 2020;216(11):153144. 10.1016/j.prp.2020.153144.

4. Zhao H, Chen S, Fu Q. Exosomes from CD133(+) cells carrying circ-ABCC1 mediate cell stemness and metastasis in colorectal cancer. J Cell Biochem. 2020;121(5-6):3286-97. 10.1002/jcb.29600.

5. Su Y, Lv X, Yin W, Zhou L, Hu Y, Zhou A, et al. CircRNA Cdr1as functions as a competitive endogenous RNA to promote hepatocellular carcinoma progression. Aging (Albany NY). 2019;11(19):8183-203. 10.18632/aging.102312.

6. Li Z, Yanfang W, Li J, Jiang P, Peng T, Chen K, et al. Tumor-released exosomal circular RNA PDE8A promotes invasive growth via the miR-338/MACC1/MET pathway in pancreatic cancer. Cancer Lett. 2018;432:237-50. 10.1016/j.canlet.2018.04.035.

7. Zhao L, Fan J, Zhang C, Zhang Z, Dong J. CircRANBP17 modulated KDM1A to regulate neuroblastoma progression by sponging miR-27b-3p. Open Med (Wars). 2023;18(1):20230672. 10.1515/med-2023-0672.

8. Zong Z-H, Du Y-P, Guan X, Chen S, Zhao Y. CircWHSC1 promotes ovarian cancer progression by regulating MUC1 and hTERT through sponging miR-145 and miR-1182. Journal of Experimental & Clinical Cancer Research. 2019;38(1):437. 10.1186/s13046-019-1437-z.

9. Ding L, Ren J, Zhang D, Li Y, Huang X, Hu Q, et al. A novel stromal lncRNA signature reprograms fibroblasts to promote the growth of oral squamous cell carcinoma via LncRNA-CAF/interleukin-33. Carcinogenesis. 2018;39(3):397-406. 10.1093/carcin/bgy006.

10. Zhang H, Wang J, Ren T, Huang Y, Yu Y, Chen C, et al. LncRNA CASC15 is Upregulated in Osteosarcoma Plasma Exosomes and CASC15 Knockdown Inhibits Osteosarcoma Progression by Regulating miR-338-3p/RAB14 Axis. Onco Targets Ther. 2020;13:12055-66. 10.2147/ott.S282053.

11. Hong J, Guo F, Lu SY, Shen C, Ma D, Zhang X, et al. F. nucleatum targets lncRNA ENO1-IT1 to promote glycolysis and oncogenesis in colorectal cancer. Gut. 2021;70(11):2123-37. 10.1136/gutjnl-2020-322780.

12. Lu Y, Chen L, Li L, Cao Y. Exosomes Derived from Brain Metastatic Breast Cancer Cells Destroy the Blood-Brain Barrier by Carrying lncRNA GS1-600G8.5. BioMed Research International. 2020;2020(1):7461727. <https://doi.org/10.1155/2020/7461727>.

13. Zhang R, Xia Y, Wang Z, Zheng J, Chen Y, Li X, et al. Serum long non coding RNA MALAT-1 protected by exosomes is up-regulated and promotes cell proliferation and migration in non-small cell lung cancer. Biochem Biophys Res Commun. 2017;490(2):406-14. 10.1016/j.bbrc.2017.06.055.

14. Wang J, Yang X, Li R, Wang L, Gu Y, Zhao Y, et al. Long non-coding RNA MYU promotes prostate cancer proliferation by mediating the miR-184/c-Myc axis. Oncol Rep. 2018;40(5):2814-25. 10.3892/or.2018.6661.

15. Yang X, Wang L, Li R, Zhao Y, Gu Y, Liu S, et al. The long non-coding RNA PCSEAT exhibits an oncogenic property in prostate cancer and functions as a competing endogenous RNA that associates with EZH2. Biochem Biophys Res Commun. 2018;502(2):262-8. 10.1016/j.bbrc.2018.05.157.

16. Yin Z, Zhou Y, Ma T, Chen S, Shi N, Zou Y, et al. Down-regulated lncRNA SBF2-AS1 in M2 macrophage-derived exosomes elevates miR-122-5p to restrict XIAP, thereby limiting pancreatic cancer development. Journal of Cellular and Molecular Medicine. 2020;24(9):5028-38. <https://doi.org/10.1111/jcmm.15125>.

17. Sánchez CA, Andahur EI, Valenzuela R, Castellón EA, Fullá JA, Ramos CG, et al. Exosomes from bulk and stem cells from human prostate cancer have a differential microRNA content that contributes cooperatively over local and pre-metastatic niche. Oncotarget. 2016;7(4):3993-4008. 10.18632/oncotarget.6540.

18. Yan T, Wang X, Wei G, Li H, Hao L, Liu Y, et al. Exosomal miR-10b-5p mediates cell communication of gastric cancer cells and fibroblasts and facilitates cell proliferation. J Cancer. 2021;12(7):2140-50. 10.7150/jca.47817.

19. Fang T, Lv H, Lv G, Li T, Wang C, Han Q, et al. Tumor-derived exosomal miR-1247-3p induces cancer-associated fibroblast activation to foster lung metastasis of liver cancer. Nat Commun. 2018;9(1):191. 10.1038/s41467-017-02583-0.

20. Geraldo MV, Yamashita AS, Kimura ET. MicroRNA miR-146b-5p regulates signal transduction of TGF-β by repressing SMAD4 in thyroid cancer. Oncogene. 2012;31(15):1910-22. 10.1038/onc.2011.381.

21. Zhang K, Li YJ, Peng LJ, Gao HF, Liu LM, Chen H. M2 macrophage-derived exosomal miR-193b-3p promotes progression and glutamine uptake of pancreatic cancer by targeting TRIM62. Biol Direct. 2023;18(1):1. 10.1186/s13062-023-00356-y.

22. Hu Y, Zhang C, Chang Q, Du J, Lu H, Guo X, et al. MicroRNA-196a-5p targeting LRP1B modulates phenotype of thyroid carcinoma cells. Endokrynol Pol. 2023;74(2):144-52. 10.5603/EP.a2023.0001.

23. Zeng Q, Zhu Z, Song L, He Z. Transferred by exosomes-derived MiR-19b-3p targets PTEN to regulate esophageal cancer cell apoptosis, migration and invasion. Biosci Rep. 2020;40(11). 10.1042/bsr20201858.

24. Abels ER, Maas SLN, Nieland L, Wei Z, Cheah PS, Tai E, et al. Glioblastoma-Associated Microglia Reprogramming Is Mediated by Functional Transfer of Extracellular miR-21. Cell Rep. 2019;28(12):3105-19.e7. 10.1016/j.celrep.2019.08.036.

25. Zhang X, Li F, Tang Y, Ren Q, Xiao B, Wan Y, et al. miR-21a in exosomes from Lewis lung carcinoma cells accelerates tumor growth through targeting PDCD4 to enhance expansion of myeloid-derived suppressor cells. Oncogene. 2020;39(40):6354-69. 10.1038/s41388-020-01406-9.

26. Liu Y, Fu W, Cao X, Li S, Xiong T, Zhang X, et al. Delivery of miR-224-5p by Exosomes from Cancer-Associated Fibroblasts Potentiates Progression of Clear Cell Renal Cell Carcinoma. Comput Math Methods Med. 2021;2021:5517747. 10.1155/2021/5517747.

27. Yokota Y, Noda T, Okumura Y, Kobayashi S, Iwagami Y, Yamada D, et al. Serum exosomal miR-638 is a prognostic marker of HCC via downregulation of VE-cadherin and ZO-1 of endothelial cells. Cancer Science. 2021;112(3):1275-88. <https://doi.org/10.1111/cas.14807>.

28. Hu X, Mu Y, Liu J, Mu X, Gao F, Chen L, et al. Exosomes Derived from Hypoxic Colorectal Cancer Cells Transfer miR-410-3p to Regulate Tumor Progression. J Cancer. 2020;11(16):4724-35. 10.7150/jca.33232.

29. Hsu Y-L, Huang M-S, Hung J-Y, Chang W-A, Tsai Y-M, Pan Y-C, et al. Bone-marrow-derived cell-released extracellular vesicle miR-92a regulates hepatic pre-metastatic niche in lung cancer. Oncogene. 2020;39(4):739-53. 10.1038/s41388-019-1024-y.

30. Liu MX, Liao J, Xie M, Gao ZK, Wang XH, Zhang Y, et al. miR-93-5p Transferred by Exosomes Promotes the Proliferation of Esophageal Cancer Cells via Intercellular Communication by Targeting PTEN. Biomed Environ Sci. 2018;31(3):171-85. 10.3967/bes2018.023.

31. Zhang C, Wei G, Zhu X, Chen X, Ma X, Hu P, et al. Exosome-Delivered circSTAU2 Inhibits the Progression of Gastric Cancer by Targeting the miR-589/CAPZA1 Axis. Int J Nanomedicine. 2023;18:127-42. 10.2147/ijn.S391872.

32. Chen W, Quan Y, Fan S, Wang H, Liang J, Huang L, et al. Exosome-transmitted circular RNA hsa_circ_0051443 suppresses hepatocellular carcinoma progression. Cancer Lett. 2020;475:119-28. 10.1016/j.canlet.2020.01.022.

33. Yang H, Qu H, Huang H, Mu Z, Mao M, Xie Q, et al. Exosomes-mediated transfer of long noncoding RNA LINC01133 represses bladder cancer progression via regulating the Wnt signaling pathway. Cell Biology International. 2021;45(7):1510-22. <https://doi.org/10.1002/cbin.11590>.

34. Zheng R, Du M, Wang X, Xu W, Liang J, Wang W, et al. Exosome-transmitted long non-coding RNA PTENP1 suppresses bladder cancer progression. Mol Cancer. 2018;17(1):143. 10.1186/s12943-018-0880-3.

35. Seol HS, Akiyama Y, Lee S-E, Shimada S, Jang SJ. Loss of miR-100 and miR-125b results in cancer stem cell properties through IGF2 upregulation in hepatocellular carcinoma. Scientific Reports. 2020;10(1):21412. 10.1038/s41598-020-77960-9.

36. Wang J, Liu Y, Li Y, Zheng X, Gan J, Wan Z, et al. Exosomal‑miR‑10a derived from colorectal cancer cells suppresses migration of human lung fibroblasts, and expression of IL‑6, IL‑8 and IL‑1β. Mol Med Rep. 2021;23(1). 10.3892/mmr.2020.11723.

37. Chang L, Gao H, Wang L, Wang N, Zhang S, Zhou X, et al. Exosomes derived from miR-1228 overexpressing bone marrow-mesenchymal stem cells promote growth of gastric cancer cells. Aging (Albany NY). 2021;13(8):11808-21. 10.18632/aging.202878.

38. Nie H, Xie X, Zhang D, Zhou Y, Li B, Li F, et al. Use of lung-specific exosomes for miRNA-126 delivery in non-small cell lung cancer. Nanoscale. 2020;12(2):877-87. 10.1039/c9nr09011h.

39. Ma H, Weng F, Wang L, Tong X, Yao Y, Li H. Extracellular vesicle-mediated delivery of miR-127-3p inhibits the proliferation and invasion of choriocarcinoma cells by targeting ITGA6. Exp Cell Res. 2022;414(2):113098. 10.1016/j.yexcr.2022.113098.

40. Yang C, Zhang G, Zhang Y, Zhang S, Li J, Liu Y. Exosome miR-134-5p restrains breast cancer progression via regulating PI3K/AKT pathway by targeting ARHGAP1. J Obstet Gynaecol Res. 2021;47(11):4037-48. 10.1111/jog.14983.

41. Chen HL, Luo YP, Lin MW, Peng XX, Liu ML, Wang YC, et al. Serum exosomal miR-16-5p functions as a tumor inhibitor and a new biomarker for PD-L1 inhibitor-dependent immunotherapy in lung adenocarcinoma by regulating PD-L1 expression. Cancer Med. 2022;11(13):2627-43. 10.1002/cam4.4638.

42. Wen H, Liu Z, Tang J, Bu L. MiR-185-5p targets RAB35 gene to regulate tumor cell-derived exosomes-mediated proliferation, migration and invasion of non-small cell lung cancer cells. Aging (Albany NY). 2021;13(17):21435-50. 10.18632/aging.203483.

43. Wang Y, Lin C. Exosomes miR-22-3p Derived from Mesenchymal Stem Cells Suppress Colorectal Cancer Cell Proliferation and Invasion by Regulating RAP2B and PI3K/AKT Pathway. J Oncol. 2021;2021:3874478. 10.1155/2021/3874478.

44. Campos-Viguri GE, Peralta-Zaragoza O, Jiménez-Wences H, Longinos-González AE, Castañón-Sánchez CA, Ramírez-Carrillo M, et al. MiR-23b-3p reduces the proliferation, migration and invasion of cervical cancer cell lines via the reduction of c-Met expression. Scientific Reports. 2020;10(1):3256. 10.1038/s41598-020-60143-x.

45. Xie H, Wang J. MicroRNA-320a-containing exosomes from human umbilical cord mesenchymal stem cells curtail proliferation and metastasis in lung cancer by binding to SOX4. J Recept Signal Transduct Res. 2022;42(3):268-78. 10.1080/10799893.2021.1918166.

46. Zuo L, Tao H, Xu H, Li C, Qiao G, Guo M, et al. Exosomes-Coated miR-34a Displays Potent Antitumor Activity in Pancreatic Cancer Both in vitro and in vivo. Drug Des Devel Ther. 2020;14:3495-507. 10.2147/dddt.S265423.

47. Vakhshiteh F, Rahmani S, Ostad SN, Madjd Z, Dinarvand R, Atyabi F. Exosomes derived from miR-34a-overexpressing mesenchymal stem cells inhibit in vitro tumor growth: A new approach for drug delivery. Life Sci. 2021;266:118871. 10.1016/j.lfs.2020.118871.

48. He Z, Li W, Zheng T, Liu D, Zhao S. Human umbilical cord mesenchymal stem cells-derived exosomes deliver microRNA-375 to downregulate ENAH and thus retard esophageal squamous cell carcinoma progression. J Exp Clin Cancer Res. 2020;39(1):140. 10.1186/s13046-020-01631-w.

49. Zhao M, Chang J, Liu R, Liu Y, Qi J, Wang Y, et al. miR-495 and miR-5688 are down-regulated in non-small cell lung cancer under hypoxia to maintain interleukin-11 expression. Cancer Communications. 2020;40(9):435-52. <https://doi.org/10.1002/cac2.12076>.

50. Pan Y, Wang X, Li Y, Yan P, Zhang H. Human umbilical cord blood mesenchymal stem cells-derived exosomal microRNA-503-3p inhibits progression of human endometrial cancer cells through downregulating MEST. Cancer Gene Therapy. 2022;29(8):1130-9. 10.1038/s41417-021-00416-3.

51. Yue S, Ye X, Zhou T, Gan D, Qian H, Fang W, et al. PGRN(-/-) TAMs-derived exosomes inhibit breast cancer cell invasion and migration and its mechanism exploration. Life Sci. 2021;264:118687. 10.1016/j.lfs.2020.118687.

52. Tubita V, Segui-Barber J, Lozano JJ, Banon-Maneus E, Rovira J, Cucchiari D, et al. Effect of immunosuppression in miRNAs from extracellular vesicles of colorectal cancer and their influence on the pre-metastatic niche. Scientific Reports. 2019;9(1):11177. 10.1038/s41598-019-47581-y.

53. Wang Z, Zhao Z, Yang Y, Luo M, Zhang M, Wang X, et al. MiR-99b-5p and miR-203a-3p Function as Tumor Suppressors by Targeting IGF-1R in Gastric Cancer. Scientific Reports. 2018;8(1):10119. 10.1038/s41598-018-27583-y.
