# Supplementary material for: Extracellular vesicles in cancer´s communication: messages we can read and how to answer
Source: Mol Cancer. 2025 Mar 19;24:86. doi: 10.1186/s12943-025-02282-1 (PMC11921637; doi:10.1186/s12943-025-02282-1)
Supplement: Supplementary file 2 — Supplementary Material 2. [file 12943_2025_2282_MOESM2_ESM.docx]

**Table S2: EV-associated proteins and lipids implying ongoing cancer-related changes**

|  | **Proteins** | | | | | | | |
| --- | --- | --- | --- | --- | --- | --- | --- | --- |
| *Protein* | | *Tissue* | *Affected systems* | *Effect* | *Marker type* | *In vitro/ in vivo* | *Ref.* |  |
| αvβ3 integrin | | Prostate cancer | *n.s.* | ↑ adhesion on vitronectin | Diagnostic | In vitro, in vivo | [1] |  |
| ADAM10, GLUT-1, GPC-1 | | Breast cancer | *n.s.* | *n.s.* | Diagnostic | In vitro | [2] |  |
| ALDH3B1, CSTB, GNAI2, IST1, KRT19, RPS2, RPL7A, RPL18, RPL28 | | Glioblastoma | *n.s.* | *n.s.* | Diagnostic | Ex vivo | [3] |  |
| ANXA6 | | Breast cancer | ↓ EGFR | ↓ apoptosis, ↑ colony formation, ↑ viability | Treatment response | In vitro, ex vivo | [4] |  |
| ApoE | | Gastric cancer | ↑ PI3K-Akt pathway | ↑ migration | Diagnostic | In vitro, in vivo | [5] |  |
| CAV1 | | Prostate cancer | *n.s.* | *n.s.* | Diagnostic | In vitro | [6] |  |
| CCT2 | | Breast cancer | *n.s.* | *n.s.* | Prognostic | In silico | [7] |  |
| HSP90AA1 | |  |  |  | Diagnostic |  |  |  |
| c-Src, Src^pY416^, IGF-IR, GRK5, GRK6, FAK, FAK^pY861^ | | Prostate cancer | *n.s.* | *n.s.* | Diagnostic | In vitro, in vivo | [8] |  |
| EDIL-3 | | Bladder cancer | *n.s.* | *n.s.* | Diagnostic | In vitro, ex vivo | [9] |  |
| ENO1 | | Hepatocellular carcinoma | ↑ integrin α6β4 ↑ FAK/Src-p38MAPK pathway | ↑ growth, ↑ metastasis | Progression | In vitro, in vivo | [10] |  |

*n.s.: not specified*

|  | **Proteins** – continued | | | | | | | |
| --- | --- | --- | --- | --- | --- | --- | --- | --- |
| *Protein* | | *Tissue* | *Affected systems* | *Effect* | *Marker type* | *In vitro/ in vivo* | *Ref.* |  |
| ENO1, FASN, PDCD6IP, XPO1 | | Prostate cancer | *n.s.* | *n.s.* | Metastatic | In vitro | [11] |  |
| GFAP, survivin | | Glioma | n.s. | ↑ local immunosuppression, ↑ proliferation, ↑ resistance to chemotherapy | Diagnostic, prognostic | Ex vivo | [12] |  |
| HMGB1 | | Gastric cancer | ↑ NF-κB pathway | ↑ migration | Diagnostic, prognostic | In vitro | [13] |  |
| HSP70, TYRP2, VLA-4 | | Melanoma | *n.s.* | *n.s.* | Prognostic | In vitro, ex vivo | [14] |  |
| MET | |  |  |  | Diagnostic |  |  |  |
| IMP1 | | Colorectal cancer | *n.s.* | *n.s.* | Diagnostic, prognostic | In vitro, in vivo | [15] |  |
| ITGA3, ITGB1 | | Prostate cancer | *n.s.* | ↑ invasion, ↑ migration | Metastatic | In vitro, ex vivo | [16] |  |
| Ku70, Ku80, Rab5c | | Rectal cancer | *n.s.* | *n.s.* | Radiotherapy resistance | In vitro | [17] |  |
| LBP | | Non-small cell lung cancer | *n.s.* | *n.s.* | Metastatic | In vitro, ex vivo | [18] |  |
| LOXL4 | | Hepatocellular carcinoma | ↑ FAK/Src pathway | ↑ invasion, ↑ metastasis, ↑ migration | Prognostic | In vitro | [19] |  |
| SORL1 | | Colorectal cancer | *n.s.* | *n.s.* | Diagnostic | In vitro | [20] |  |
| TACSTD2 | | Bladder cancer | *n.s.* | *n.s.* | Diagnostic | Ex vivo | [21] |  |
| TPX2 | | Non-small cell lung cancer | ↑ WNT/β-catenin signaling pathway | ↑ invasion, ↑ metastasis, ↑ migration, ↓ sensitivity to docetaxel | Prognostic | In vitro, in vivo | [22] |  |

*n.s.: not specified*

| **Lipids** | | | | |  |
| --- | --- | --- | --- | --- | --- |
| *Lipid* | *Tissue* | *Marker type* | *In vitro/ in vivo* | *Ref.* |  |
| Cardiolipins, sphingosines | Hepatocellular cancer | Diagnostic | Ex vivo | [23] |  |
| ↑ ceramide (d18:1_16:0), ↑ ceramide (d18:1_18:0), ↓ sphingomyelins | | Hepatocellular carcinoma, intrahepatic cholangiocarcinoma | Diagnostic | Ex vivo | [24] |
| Cholesterol ester, zymosterol | Ovarian cancer | Diagnostic | In vitro | [25] |  |
| Glycerophospholipids, sphingolipids, sterol lipids | Prostate cancer | Diagnostic, metastatic | In vitro | [26] |  |
| Hexosylceramide, lactosylceramide | Prostate cancer | Diagnostic | Ex vivo | [27] |  |
| ↑ hexosylceramide d18:1/24:0, ↑ hexosylceramide d18:1/24:1, ↑ phosphatidylcholine 34:1, ↑ phosphatidylethanolamine 36:2, ↑ sphingomyelin d18:1/16:0 | Colorectal cancer | Diagnostic | In vitro, ex vivo | [28] |  |
| ↑ ceramide d18:1/24:1, ↓ phosphatidylethanolamine 34:2, ↓ phosphatidylethanolamine 36:2, ↓ plasmalogen 16:0/20:4, |  | Metastatic |  |  |  |
| Phosphatidylethanolamine 34:1, phosphatidylethanolamine (16:0/18:1) | Pancreatic cancer | Prognostic | Ex vivo | [29] |  |

1. Singh A, Fedele C, Lu H, Nevalainen MT, Keen JH, Languino LR. Exosome-mediated Transfer of αvβ3 Integrin from Tumorigenic to Nontumorigenic Cells Promotes a Migratory Phenotype. Mol Cancer Res. 2016;14(11):1136-46. 10.1158/1541-7786.Mcr-16-0058.

2. Risha Y, Minic Z, Ghobadloo SM, Berezovski MV. The proteomic analysis of breast cell line exosomes reveals disease patterns and potential biomarkers. Scientific Reports. 2020;10(1):13572. 10.1038/s41598-020-70393-4.

3. Hallal SM, Tűzesi Á, Sida LA, Xian E, Madani D, Muralidharan K, et al. Glioblastoma biomarkers in urinary extracellular vesicles reveal the potential for a ‘liquid gold’ biopsy. British Journal of Cancer. 2024;130(5):836-51. 10.1038/s41416-023-02548-9.

4. Li T, Tao Z, Zhu Y, Liu X, Wang L, Du Y, et al. Exosomal annexin A6 induces gemcitabine resistance by inhibiting ubiquitination and degradation of EGFR in triple-negative breast cancer. Cell Death Dis. 2021;12(7):684. 10.1038/s41419-021-03963-7.

5. Zheng P, Luo Q, Wang W, Li J, Wang T, Wang P, et al. Tumor-associated macrophages-derived exosomes promote the migration of gastric cancer cells by transfer of functional Apolipoprotein E. Cell Death Dis. 2018;9(4):434. 10.1038/s41419-018-0465-5.

6. Ariotti N, Wu Y, Okano S, Gambin Y, Follett J, Rae J, et al. An inverted CAV1 (caveolin 1) topology defines novel autophagy-dependent exosome secretion from prostate cancer cells. Autophagy. 2021;17(9):2200-16. 10.1080/15548627.2020.1820787.

7. Alagundagi DB, Ghate SD, Rajendra VKJ, Gollapalli P, Shetty VV, D'Souza C, et al. Exploring breast cancer exosomes for novel biomarkers of potential diagnostic and prognostic importance. 3 Biotech. 2023;13(1):7. 10.1007/s13205-022-03422-w.

8. DeRita RM, Zerlanko B, Singh A, Lu H, Iozzo RV, Benovic JL, et al. c-Src, Insulin-Like Growth Factor I Receptor, G-Protein-Coupled Receptor Kinases and Focal Adhesion Kinase are Enriched Into Prostate Cancer Cell Exosomes. J Cell Biochem. 2017;118(1):66-73. 10.1002/jcb.25611.

9. Beckham CJ, Olsen J, Yin PN, Wu CH, Ting HJ, Hagen FK, et al. Bladder cancer exosomes contain EDIL-3/Del1 and facilitate cancer progression. J Urol. 2014;192(2):583-92. 10.1016/j.juro.2014.02.035.

10. Jiang K, Dong C, Yin Z, Li R, Mao J, Wang C, et al. Exosome-derived ENO1 regulates integrin α6β4 expression and promotes hepatocellular carcinoma growth and metastasis. Cell Death & Disease. 2020;11(11):972. 10.1038/s41419-020-03179-1.

11. Duijvesz D, Burnum-Johnson KE, Gritsenko MA, Hoogland AM, Vredenbregt-van den Berg MS, Willemsen R, et al. Proteomic profiling of exosomes leads to the identification of novel biomarkers for prostate cancer. PLoS One. 2013;8(12):e82589. 10.1371/journal.pone.0082589.

12. Galbo PM, Jr., Ciesielski MJ, Figel S, Maguire O, Qiu J, Wiltsie L, et al. Circulating CD9+/GFAP+/survivin+ exosomes in malignant glioma patients following survivin vaccination. Oncotarget. 2017;8(70):114722-35. 10.18632/oncotarget.21773.

13. Zhang X, Shi H, Yuan X, Jiang P, Qian H, Xu W. Tumor-derived exosomes induce N2 polarization of neutrophils to promote gastric cancer cell migration. Molecular Cancer. 2018;17(1):146. 10.1186/s12943-018-0898-6.

14. Peinado H, Alečković M, Lavotshkin S, Matei I, Costa-Silva B, Moreno-Bueno G, et al. Melanoma exosomes educate bone marrow progenitor cells toward a pro-metastatic phenotype through MET. Nat Med. 2012;18(6):883-91. 10.1038/nm.2753.

15. Kuhn M, Zhang Y, Favate J, Morita M, Blucher A, Das S, et al. IMP1/IGF2BP1 in human colorectal cancer extracellular vesicles. Am J Physiol Gastrointest Liver Physiol. 2022;323(6):G571-g85. 10.1152/ajpgi.00121.2022.

16. Bijnsdorp IV, Geldof AA, Lavaei M, Piersma SR, van Moorselaar RJ, Jimenez CR. Exosomal ITGA3 interferes with non-cancerous prostate cell functions and is increased in urine exosomes of metastatic prostate cancer patients. J Extracell Vesicles. 2013;2. 10.3402/jev.v2i0.22097.

17. Martins VR, Baptistella AR, Silva PP, Cassinela EK, Marchi F, Leme AF, et al. Abstract 1567: Ku70, Ku80 and Rab5C as biomarkers for radiotherapy resistance in rectal tumors during neoadjuvant therapy. Cancer Research. 2018;78(13_Supplement):1567-. 10.1158/1538-7445.Am2018-1567.

18. Wang N, Song X, Liu L, Niu L, Wang X, Song X, et al. Circulating exosomes contain protein biomarkers of metastatic non-small-cell lung cancer. Cancer Sci. 2018;109(5):1701-9. 10.1111/cas.13581.

19. Li R, Wang Y, Zhang X, Feng M, Ma J, Li J, et al. Exosome-mediated secretion of LOXL4 promotes hepatocellular carcinoma cell invasion and metastasis. Molecular Cancer. 2019;18(1):18. 10.1186/s12943-019-0948-8.

20. Li P, Chen J, Chen Y, Song S, Huang X, Yang Y, et al. Construction of Exosome SORL1 Detection Platform Based on 3D Porous Microfluidic Chip and its Application in Early Diagnosis of Colorectal Cancer. Small. 2023;19(20):e2207381. 10.1002/smll.202207381.

21. Chen C-L, Lai Y-F, Tang P, Chien K-Y, Yu J-S, Tsai C-H, et al. Comparative and Targeted Proteomic Analyses of Urinary Microparticles from Bladder Cancer and Hernia Patients. Journal of Proteome Research. 2012;11(12):5611-29. 10.1021/pr3008732.

22. Hu J, He Q, Tian T, Chang N, Qian L. Transmission of Exosomal TPX2 Promotes Metastasis and Resistance of NSCLC Cells to Docetaxel. Onco Targets Ther. 2023;16:197-210. 10.2147/ott.S401454.

23. Sanchez JI, Jiao J, Kwan S-Y, Veillon L, Warmoes MO, Tan L, et al. Lipidomic Profiles of Plasma Exosomes Identify Candidate Biomarkers for Early Detection of Hepatocellular Carcinoma in Patients with Cirrhosis. Cancer Prevention Research. 2021;14(10):955-62. 10.1158/1940-6207.Capr-20-0612.

24. Yang K, Fu W, Deng M, Li X, Wu M, Wang Y. The sphingolipids change in exosomes from cancer patients and association between exosome release and sphingolipids level based on a pseudotargeted lipidomics method. Analytica Chimica Acta. 2024;1305:342527. <https://doi.org/10.1016/j.aca.2024.342527>.

25. Cheng L, Zhang K, Qing Y, Li D, Cui M, Jin P, et al. Proteomic and lipidomic analysis of exosomes derived from ovarian cancer cells and ovarian surface epithelial cells. Journal of Ovarian Research. 2020;13(1):9. 10.1186/s13048-020-0609-y.

26. Brzozowski JS, Jankowski H, Bond DR, McCague SB, Munro BR, Predebon MJ, et al. Lipidomic profiling of extracellular vesicles derived from prostate and prostate cancer cell lines. Lipids in Health and Disease. 2018;17(1):211. 10.1186/s12944-018-0854-x.

27. Skotland T, Ekroos K, Kauhanen D, Simolin H, Seierstad T, Berge V, et al. Molecular lipid species in urinary exosomes as potential prostate cancer biomarkers. European Journal of Cancer. 2017;70:122-32. <https://doi.org/10.1016/j.ejca.2016.10.011>.

28. Elmallah MIY, Ortega-Deballon P, Hermite L, Pais-De-Barros J-P, Gobbo J, Garrido C. Lipidomic profiling of exosomes from colorectal cancer cells and patients reveals potential biomarkers. Molecular Oncology. 2022;16(14):2710-8. <https://doi.org/10.1002/1878-0261.13223>.

29. Tao L, Zhou J, Yuan C, Zhang L, Li D, Si D, et al. Metabolomics identifies serum and exosomes metabolite markers of pancreatic cancer. Metabolomics. 2019;15(6):86. 10.1007/s11306-019-1550-1.
