# Supplementary material for: Extracellular vesicles in cancer´s communication: messages we can read and how to answer
Source: Mol Cancer. 2025 Mar 19;24:86. doi: 10.1186/s12943-025-02282-1 (PMC11921637; doi:10.1186/s12943-025-02282-1)
Supplement: Supplementary file 3 — Supplementary Material 3. [file 12943_2025_2282_MOESM3_ESM.docx]

**Table S3: EV-associated RNAs and proteins affecting angiogenesis**

| **Protumorigenic effect** | | | | | |
| --- | --- | --- | --- | --- | --- |
| *RNA* | *Tissue* | *Affected systems* | *Effect* | *In vitro/in vivo* | *Ref.* |
| circR-100338 | Hepatocellular carcinoma | *n.s.* | ↑ tumor progression (cancer cell proliferation, angiogenesis) | In vitro, in vivo | [1] |
| lncR-CCAT2 | Glioma | ↑ Bcl-2 expression, ↓ Bax and caspase-3 expression | ↑ angiogenesis, ↓ apoptosis | In vitro, in vivo | [2] |
| miR-3157-3p | Non-small cell lung cancer | ↓ TIMP2/KLF2 | ↑ angiogenesis, ↑ metastasis, ↑ vascular permeability | In vitro, in vivo | [3] |
| miR-9-5p | Glioma | *n.s.* | ↑ angiogenesis | In vitro | [4] |
| *Protein* | *Tissue* | *Affected systems* | *Effect* | *In vitro/in vivo* | *Ref.* |
| LRG1 | Non-small cell lung cancer | ↑ TGF-β pathway | ↑ angiogenesis | In vitro | [5] |
| M6PR | Esophageal squamous cell cancer | *n.s.* | ↑ angiogenesis | In vitro | [6] |
| **Suppressive effect** | | | | | |
| *RNA* | *Tissue* | *Affected systems* | *Effect* | *In vitro/in vivo* | *Ref.* |
| miR-125a-3p | Nasopharyngeal cancer | ↓ TAZ | ↓ angiogenesis, ↓ vasculogenic mimicry | In vitro, in vivo | [7] |

*n.s.: not specified*

1. Huang X-Y, Huang Z-L, Huang J, Xu B, Huang X-Y, Xu Y-H, et al. Exosomal circRNA-100338 promotes hepatocellular carcinoma metastasis via enhancing invasiveness and angiogenesis. Journal of Experimental & Clinical Cancer Research. 2020;39(1):20. 10.1186/s13046-020-1529-9.

2. Lang HL, Hu GW, Zhang B, Kuang W, Chen Y, Wu L, et al. Glioma cells enhance angiogenesis and inhibit endothelial cell apoptosis through the release of exosomes that contain long non-coding RNA CCAT2. Oncol Rep. 2017;38(2):785-98. 10.3892/or.2017.5742.

3. Ma Z, Wei K, Yang F, Guo Z, Pan C, He Y, et al. Tumor-derived exosomal miR-3157-3p promotes angiogenesis, vascular permeability and metastasis by targeting TIMP/KLF2 in non-small cell lung cancer. Cell Death Dis. 2021;12(9):840. 10.1038/s41419-021-04037-4.

4. Lucero R, Zappulli V, Sammarco A, Murillo OD, Cheah PS, Srinivasan S, et al. Glioma-Derived miRNA-Containing Extracellular Vesicles Induce Angiogenesis by Reprogramming Brain Endothelial Cells. Cell Rep. 2020;30(7):2065-74.e4. 10.1016/j.celrep.2020.01.073.

5. Li Z, Zeng C, Nong Q, Long F, Liu J, Mu Z, et al. Exosomal Leucine-Rich-Alpha2-Glycoprotein 1 Derived from Non-Small-Cell Lung Cancer Cells Promotes Angiogenesis via TGF-β Signal Pathway. Mol Ther Oncolytics. 2019;14:313-22. 10.1016/j.omto.2019.08.001.

6. Yan D, Cui D, Zhu Y, Chan C, Choi C, Liu T, et al. M6PR- and EphB4-Rich Exosomes Secreted by Serglycin-Overexpressing Esophageal Cancer Cells Promote Cancer Progression. International Journal of Biological Sciences. 2023;19:625-40. 10.7150/ijbs.79875.

7. Wan F, Zhang H, Hu J, Chen L, Geng S, Kong L, et al. Mesenchymal Stem Cells Inhibits Migration and Vasculogenic Mimicry in Nasopharyngeal Carcinoma Via Exosomal MiR-125a. Front Oncol. 2022;12:781979. 10.3389/fonc.2022.781979.
