# Supplementary material for: Extracellular vesicles in cancer´s communication: messages we can read and how to answer
Source: Mol Cancer. 2025 Mar 19;24:86. doi: 10.1186/s12943-025-02282-1 (PMC11921637; doi:10.1186/s12943-025-02282-1)
Supplement: Supplementary file 4 — Supplementary Material 4. [file 12943_2025_2282_MOESM4_ESM.docx]

**Table S4: EV-associated RNAs and proteins affecting metastasis**

| **Metastasis promoting** | | | | | |
| --- | --- | --- | --- | --- | --- |
| *RNA* | *Tissue* | *Affected systems* | *Effect* | *In vitro/in vivo* | *Ref.* |
| circR-0000284 | Cholangiocarcinoma | ↓ miR-637 | ↑ invasion, ↑ migration, ↑ proliferation | In vitro, in vivo | [1] |
| circR-00448516 | Prostate cancer | ↓ miR-29a-3p | ↑ metastasis, ↑ proliferation | In vitro, in vivo | [2] |
| circR-IARS | Pancreatic ductal adenocarcinoma | ↓ miRNA-122, ZO-1 ↑ RhoA, RhoA-GTP, F-actin | ↑ invasion, ↑ metastasis | In vitro, in vivo | [3] |
| circR-NRIP1 | Gastric cancer | ↓ miR-149-5p ↑ AKT1/mTOR pathway | ↑ metastasis | In vitro, in vivo | [4] |
| circR-PRMT5 | Urothelial carcinoma | ↓ miR-30c, E-cadherin ↑ SNAIL1 | ↑ epithelial-mesenchymal transition, ↑ metastasis | In vitro, ex vivo | [5] |
| circR-RanGAP1 | Gastric cancer | ↓ miR-877-3p ↑ VEGFA | ↑ invasion ↑ migration | In vitro, in vivo | [6] |
| lncR-91H | Colorectal cancer | interacts with HNRNPK | ↑ invasion, ↑ migration, ↑ proliferation | In vitro, in vivo | [7] |
| lncR-FAL1 | Hepatocellular carcinoma | ↓ miR-1236 | ↑ metastasis, ↑ proliferation | In vitro, ex vivo | [8] |
| lncR-HISLA | Bladder cancer | ↑ Wnt/β-catenin | ↑ epithelial-mesenchymal transition, ↑ invasion, ↑ migration | In vitro, in vivo | [9] |
| lncR-HULC | Pancreatic ductal adenocarcinoma | *n.s.* | ↑ epithelial-mesenchymal transition, ↑ invasion, ↑ migration | In vitro, in vivo | [10] |
| lncR MRPL23-AS1 | Salivary adenoid cystic carcinoma | ↓ E-cadherin | ↑ epithelial-mesenchymal transition, ↑ lung metastasis, ↑ microvascular permeability | In vitro, in vivo | [11] |

*n.s.: not specified*

| **Metastasis promoting** - continued | | | | | |
| --- | --- | --- | --- | --- | --- |
| *RNA* | *Tissue* | *Affected systems* | *Effect* | *In vitro/in vivo* | *Ref.* |
| lncR- Sox2ot | Pancreatic ductal adenocarcinoma | ↓ miR-200, E-cadherin ↑ Sox2, N-cadherin, vimentin | ↑ epithelial-mesenchymal transition, ↑ invasion, ↑ metastasis, ↑ stemness | In vitro, in vivo | [12] |
| lncR- TUC339 | Hepatocellular cancer | *n.s.* | ↑ adhesion, ↑ cancer cell growth, ↑ invasion | In vitro | [13] |
| lncR-ZFAS1 | Gastric cancer | ↑ cyclin D1, Bcl2, | ↑ migration, ↑ proliferation | In vitro | [14] |
| miR-105 | Breast cancer | ↓ ZO1 | ↑ metastasis, ↑ migration, ↑ vascular permeability | In vitro | [15] |
| miR-122 | Breast cancer | ↓ PKM, CS, GLUT1 | ↓ glucose uptake in non-tumor cells in pre-metastatic niche, ↑ metastasis | In vitro, in vivo | [16] |
| miR-1246, miR-27a-3p, miR-92b-3p | Colorectal cancer | ↓ GSK3β, ↑ Wnt/β-catenin | ↑ metastasis, ↑ migration | In vitro, in vivo | [17] |
| miR-1290 | Prostate cancer | ↓ GSK3β/β-catenin signalling | ↑ metastasis, ↑ tumor growth | In vitro, in vivo | [18] |
| miR-146a | Breast cancer | ↓ TXNIP, ↑ Wnt signaling | ↑ invasion, ↑ migration | In vitro, in vivo | [19] |
| miR-155-5p | Colon cancer | ↓ BRG1 | ↑ invasion, ↑ migration | In vitro, in vivo | [20] |
| miR-192, miR-215 | Gastric cancer | ↓ Rab11-FIP2 | ↑ invasion, ↑lymphatic metastasis, ↑ migration, ↑ proliferation | In vitro | [21, 22] |

*n.s.: not specified*

| **Metastasis promoting** - continued | | | | | | |
| --- | --- | --- | --- | --- | --- | --- |
| *RNA* | | *Tissue* | *Affected systems* | *Effect* | *In vitro/in vivo* | *Ref.* |
| miR-19a | Breast cancer | ↓ PTEN, ↑ NF-κB, AKT | ↑ osteolytic bone metastasis, prognostic biomarker | In vitro, in vivo | [23] |  |
| miR-24-3p | Breast cancer | ↑ TWIST1/IL-3Rα | ↑ metastatic lesions | In vivo | [24] |  |
| miR-210 | Non-small cell lung cancer | ↑ UPF1, PTEN/PI3K/AKT pathway | ↑ epithelial-mesenchymal transition, ↑ invasion, ↑ migration, ↑ proliferation | In vitro, in vivo | [25] |  |
| miR-301a-3p | Gastric cancer (hypoxic conditions) | ↓ PDH3 | ↑ epithelial-mesenchymal transition, ↑ metastasis, ↑ progression | In vitro, in vivo | [26] |  |
| miR-3473b | Lung cancer | ↓ NFKBID, ↑ NF-κB | ↑ intrapulmonary colonization | In vitro | [27] |  |
| miR-375 | Prostate cancer | ↓ PTPN4  ↑ STAT3 | ↑ epithelial-mesenchymal transition | In vitro | [28] |  |
| miR-375-3p | Small cell lung cancer | ↓ claudin-1 | ↑ blood vessel permeability, ↑ metastasis, ↑ permeability of vascular endothelial cells, ↑ transendothelial migration | In vitro, in vivo | [29] |  |
| miR-378a-3p | Prostate cancer | ↑ Dyrk1a/Nfatc1/Angptl2 axis | ↑ proliferation, ↑ epithelial-mesenchymal transition, ↑ osteolysis | In vitro, in vivo | [30] |  |
| miR-486-5p | Rhabdomyosarcoma | mediates PAX3-FOXO1 paracrine effects | ↑ invasion, ↑ migration, | In vitro | [31] |  |
| miR-629-5p | Lung adenocarcinoma | ↓ PPWD1, CELSR1 | ↑ endothelial monolayer permeability, ↑ invasion | In vitro, in vivo | [32] |  |
| miR-939 | Breast cancer | ↓ VE-cadherin | ↑ trans-endothelial migration | In vitro | [33] |  |

| **Metastasis promoting** - continued | | | | | |
| --- | --- | --- | --- | --- | --- |
| *Protein* | *Tissue* | *Affected systems* | *Effect* | *In vitro/in vivo* | *Ref.* |
| ENO1 | Hepatocellular carcinoma | ↑ integrin α6β4 ↑ FAK/Src-p38MAPK pathway | ↑ growth, ↑ metastasis | In vitro, in vivo | [34] |
| GRP78 | Colon cancer | *n.s.* | ↑ migration, ↑ proliferation, ↑ stemness | In vitro | [35] |
| LOXL4 | Hepatocellular carcinoma | ↑ FAK/Src pathway | ↑ invasion, ↑ metastasis, ↑ migration | In vitro | [36] |
| PKM2 | Prostate cancer | ↑ CXCL12 | ↑ premetastatic niche formation, ↑ seeding in bone marrow | In vitro, ex vivo | [37] |
| TPX2 | Non-small cell lung cancer | ↑ WNT/β-catenin signaling pathway | ↑ invasion, ↑ metastasis, ↑ migration | In vitro, in vivo | [38] |

| **Metastasis suppressing** | | | | | |
| --- | --- | --- | --- | --- | --- |
| *RNA* | *Tissue* | *Affected systems* | *Effect* | *In vitro/in vivo* | *Ref.* |
| let-71-5p | Colon cancer | ↓ KLK6 | ↑ apoptosis, ↓ metastasis, ↓ proliferation | In vitro, in vivo | [39] |
| lncR-MMP2-2 | Lung cancer | ↑ MMP2 | ↓ invasion, ↓ migration | In vitro | [40] |
| lncR-NONHSAT105177 | Pancreatic ductal adenocarcinoma | ↓ CLU pathway | ↓ epithelial-mesenchymal transition, ↓ migration, ↓ proliferation | In vitro, in vivo | [41] |
| miR-124 | Pancreatic cancer | ↓ EZH2 | ↑ apoptosis, ↓ epithelial-mesenchymal transition, ↓ invasion, ↓ migration | In vitro, in vivo | [42] |
| miR-126-3p | Liver cancer | ↓ ADAM9 | ↓ invasion, ↓ migration | In vitro | [43] |
| miR-141-3p | Prostate cancer | ↓ NF-κB signaling | ↓ metastasis | In vivo | [44] |
| miR-145 | Breast cancer | ↑ ROCK1, TP53 ↓ ERBB2, MMP9 | ↑ apoptosis, ↓ metastasis | In vitro | [45] |
| miR-148b | Endometrial cancer | ↓ DNMT1 | ↓ epithelial-mesenchymal transition, ↓ invasion, ↓ migration | In vivo, in vitro | [46] |
| miR-196b | Colorectal cancer | ↓ HOXA9, GALNT5  ↓ SOCS1, SOCS3 ↑STAT3 signaling | ↓ metastasis, ↓ migration | In vitro, in vivo | [47, 48] |
| miR-200b-3p | Colorectal cancer | ↓ ZEB1, E2F3 | ↓ invasion, ↓ migration, ↑ sensitivity to 5-fluorouracil,  ↓ stemness | In vitro, in vivo | [49] |
| miR-29c-3p | CAF in ovarian cancer | ↓ MMP2 | ↓ metastasis | In vitro, in vivo | [50] |
| miR-34c-3p | Non-small cell lung cancer | ↑ integrin α2β1 | ↓ invasion, ↓ metastasis | In vitro, in vivo | [51] |

1. Wang S, Hu Y, Lv X, Li B, Gu D, Li Y, et al. Circ-0000284 arouses malignant phenotype of cholangiocarcinoma cells and regulates the biological functions of peripheral cells through cellular communication. Clin Sci (Lond). 2019;133(18):1935-53. 10.1042/cs20190589.

2. Li T, Sun X, Chen L. Exosome circ_0044516 promotes prostate cancer cell proliferation and metastasis as a potential biomarker. J Cell Biochem. 2020;121(3):2118-26. 10.1002/jcb.28239.

3. Li J, Li Z, Jiang P, Peng M, Zhang X, Chen K, et al. Circular RNA IARS (circ-IARS) secreted by pancreatic cancer cells and located within exosomes regulates endothelial monolayer permeability to promote tumor metastasis. J Exp Clin Cancer Res. 2018;37(1):177. 10.1186/s13046-018-0822-3.

4. Zhang X, Wang S, Wang H, Cao J, Huang X, Chen Z, et al. Circular RNA circNRIP1 acts as a microRNA-149-5p sponge to promote gastric cancer progression via the AKT1/mTOR pathway. Mol Cancer. 2019;18(1):20. 10.1186/s12943-018-0935-5.

5. Chen X, Chen RX, Wei WS, Li YH, Feng ZH, Tan L, et al. PRMT5 Circular RNA Promotes Metastasis of Urothelial Carcinoma of the Bladder through Sponging miR-30c to Induce Epithelial-Mesenchymal Transition. Clin Cancer Res. 2018;24(24):6319-30. 10.1158/1078-0432.Ccr-18-1270.

6. Lu J, Wang YH, Yoon C, Huang XY, Xu Y, Xie JW, et al. Circular RNA circ-RanGAP1 regulates VEGFA expression by targeting miR-877-3p to facilitate gastric cancer invasion and metastasis. Cancer Lett. 2020;471:38-48. 10.1016/j.canlet.2019.11.038.

7. Gao T, Liu X, He B, Nie Z, Zhu C, Zhang P, et al. Exosomal lncRNA 91H is associated with poor development in colorectal cancer by modifying HNRNPK expression. Cancer Cell Int. 2018;18:11. 10.1186/s12935-018-0506-2.

8. Li B, Mao R, Liu C, Zhang W, Tang Y, Guo Z. LncRNA FAL1 promotes cell proliferation and migration by acting as a CeRNA of miR-1236 in hepatocellular carcinoma cells. Life Sci. 2018;197:122-9. 10.1016/j.lfs.2018.02.006.

9. Guo Y, Li Z, Sun W, Gao W, Liang Y, Mei Z, et al. M2 Tumor Associate Macrophage- (TAM-) Derived lncRNA HISLA Promotes EMT Potential in Bladder Cancer. Journal of Oncology. 2022;2022(1):8268719. <https://doi.org/10.1155/2022/8268719>.

10. Takahashi K, Ota Y, Kogure T, Suzuki Y, Iwamoto H, Yamakita K, et al. Circulating extracellular vesicle-encapsulated HULC is a potential biomarker for human pancreatic cancer. Cancer Science. 2020;111(1):98-111. <https://doi.org/10.1111/cas.14232>.

11. Chen CW, Fu M, Du ZH, Zhao F, Yang WW, Xu LH, et al. Long Noncoding RNA MRPL23-AS1 Promotes Adenoid Cystic Carcinoma Lung Metastasis. Cancer Res. 2020;80(11):2273-85. 10.1158/0008-5472.Can-19-0819.

12. Li Z, Jiang P, Li J, Peng M, Zhao X, Zhang X, et al. Tumor-derived exosomal lnc-Sox2ot promotes EMT and stemness by acting as a ceRNA in pancreatic ductal adenocarcinoma. Oncogene. 2018;37(28):3822-38. 10.1038/s41388-018-0237-9.

13. Kogure T, Yan IK, Lin WL, Patel T. Extracellular Vesicle-Mediated Transfer of a Novel Long Noncoding RNA TUC339: A Mechanism of Intercellular Signaling in Human Hepatocellular Cancer. Genes Cancer. 2013;4(7-8):261-72. 10.1177/1947601913499020.

14. Pan L, Liang W, Fu M, Huang ZH, Li X, Zhang W, et al. Exosomes-mediated transfer of long noncoding RNA ZFAS1 promotes gastric cancer progression. J Cancer Res Clin Oncol. 2017;143(6):991-1004. 10.1007/s00432-017-2361-2.

15. Zhou W, Fong MY, Min Y, Somlo G, Liu L, Palomares MR, et al. Cancer-secreted miR-105 destroys vascular endothelial barriers to promote metastasis. Cancer Cell. 2014;25(4):501-15. 10.1016/j.ccr.2014.03.007.

16. Fong MY, Zhou W, Liu L, Alontaga AY, Chandra M, Ashby J, et al. Breast-cancer-secreted miR-122 reprograms glucose metabolism in premetastatic niche to promote metastasis. Nat Cell Biol. 2015;17(2):183-94. 10.1038/ncb3094.

17. Guo S, Chen J, Chen F, Zeng Q, Liu WL, Zhang G. Exosomes derived from Fusobacterium nucleatum-infected colorectal cancer cells facilitate tumour metastasis by selectively carrying miR-1246/92b-3p/27a-3p and CXCL16. Gut. 2020;10.1136/gutjnl-2020-321187. 10.1136/gutjnl-2020-321187.

18. Wang S, Du P, Cao Y, Ma J, Yang X, Yu Z, et al. Cancer associated fibroblasts secreted exosomal miR-1290 contributes to prostate cancer cell growth and metastasis via targeting GSK3β. Cell Death Discovery. 2022;8(1):371. 10.1038/s41420-022-01163-6.

19. Yang SS, Ma S, Dou H, Liu F, Zhang SY, Jiang C, et al. Breast cancer-derived exosomes regulate cell invasion and metastasis in breast cancer via miR-146a to activate cancer associated fibroblasts in tumor microenvironment. Exp Cell Res. 2020;391(2):111983. 10.1016/j.yexcr.2020.111983.

20. Lan J, Sun L, Xu F, Liu L, Hu F, Song D, et al. M2 Macrophage-Derived Exosomes Promote Cell Migration and Invasion in Colon Cancer. Cancer Res. 2019;79(1):146-58. 10.1158/0008-5472.Can-18-0014.

21. Zhang X, Peng Y, Huang Y, Deng S, Feng X, Hou G, et al. Inhibition of the miR-192/215–Rab11-FIP2 axis suppresses human gastric cancer progression. Cell Death & Disease. 2018;9(7):778. 10.1038/s41419-018-0785-5.

22. He J, Wu J, Dong S, Xu J, Wang J, Zhou X, et al. Exosome-Encapsulated miR-31, miR-192, and miR-375 Serve as Clinical Biomarkers of Gastric Cancer. Journal of Oncology. 2023;2023(1):7335456. <https://doi.org/10.1155/2023/7335456>.

23. Wu K, Feng J, Lyu F, Xing F, Sharma S, Liu Y, et al. Exosomal miR-19a and IBSP cooperate to induce osteolytic bone metastasis of estrogen receptor-positive breast cancer. Nat Commun. 2021;12(1):5196. 10.1038/s41467-021-25473-y.

24. Lopatina T, Grange C, Cavallari C, Navarro-Tableros V, Lombardo G, Rosso A, et al. Targeting IL-3Rα on tumor-derived endothelial cells blunts metastatic spread of triple-negative breast cancer via extracellular vesicle reprogramming. Oncogenesis. 2020;9(10):90. 10.1038/s41389-020-00274-y.

25. Yang F, Yan Y, Yang Y, Hong X, Wang M, Yang Z, et al. MiR-210 in exosomes derived from CAFs promotes non-small cell lung cancer migration and invasion through PTEN/PI3K/AKT pathway. Cell Signal. 2020;73:109675. 10.1016/j.cellsig.2020.109675.

26. Xia X, Wang S, Ni B, Xing S, Cao H, Zhang Z, et al. Hypoxic gastric cancer-derived exosomes promote progression and metastasis via MiR-301a-3p/PHD3/HIF-1α positive feedback loop. Oncogene. 2020;39(39):6231-44. 10.1038/s41388-020-01425-6.

27. Du C, Duan X, Yao X, Wan J, Cheng Y, Wang Y, et al. Tumour-derived exosomal miR-3473b promotes lung tumour cell intrapulmonary colonization by activating the nuclear factor-κB of local fibroblasts. Journal of Cellular and Molecular Medicine. 2020;24(14):7802-13. <https://doi.org/10.1111/jcmm.15411>.

28. Gan J, Liu S, Zhang Y, He L, Bai L, Liao R, et al. MicroRNA-375 is a therapeutic target for castration-resistant prostate cancer through the PTPN4/STAT3 axis. Experimental & Molecular Medicine. 2022;54(8):1290-305. 10.1038/s12276-022-00837-6.

29. Mao S, Zheng S, Lu Z, Wang X, Wang Y, Zhang G, et al. Exosomal miR-375-3p breaks vascular barrier and promotes small cell lung cancer metastasis by targeting claudin-1. Transl Lung Cancer Res. 2021;10(7):3155-72. 10.21037/tlcr-21-356.

30. Wang J, Du X, Wang X, Xiao H, Jing N, Xue W, et al. Tumor-derived miR-378a-3p-containing extracellular vesicles promote osteolysis by activating the Dyrk1a/Nfatc1/Angptl2 axis for bone metastasis. Cancer Lett. 2022;526:76-90. 10.1016/j.canlet.2021.11.017.

31. Ghamloush F, Ghayad SE, Rammal G, Fahs A, Ayoub AJ, Merabi Z, et al. The PAX3-FOXO1 oncogene alters exosome miRNA content and leads to paracrine effects mediated by exosomal miR-486. Scientific Reports. 2019;9(1):14242. 10.1038/s41598-019-50592-4.

32. Li Y, Zhang H, Fan L, Mou J, Yin Y, Peng C, et al. MiR-629-5p promotes the invasion of lung adenocarcinoma via increasing both tumor cell invasion and endothelial cell permeability. Oncogene. 2020;39(17):3473-88. 10.1038/s41388-020-1228-1.

33. Di Modica M, Regondi V, Sandri M, Iorio MV, Zanetti A, Tagliabue E, et al. Breast cancer-secreted miR-939 downregulates VE-cadherin and destroys the barrier function of endothelial monolayers. Cancer Lett. 2017;384:94-100. 10.1016/j.canlet.2016.09.013.

34. Jiang K, Dong C, Yin Z, Li R, Mao J, Wang C, et al. Exosome-derived ENO1 regulates integrin α6β4 expression and promotes hepatocellular carcinoma growth and metastasis. Cell Death & Disease. 2020;11(11):972. 10.1038/s41419-020-03179-1.

35. Tsurusawa N, Iha K, Sato A, Tsai HY, Sonoda H, Watabe S, et al. Ultrasensitive Detection of GRP78 in Exosomes and Observation of Migration and Proliferation of Cancer Cells by Application of GRP78-Containing Exosomes. Cancers (Basel). 2022;14(16). 10.3390/cancers14163887.

36. Li R, Wang Y, Zhang X, Feng M, Ma J, Li J, et al. Exosome-mediated secretion of LOXL4 promotes hepatocellular carcinoma cell invasion and metastasis. Molecular Cancer. 2019;18(1):18. 10.1186/s12943-019-0948-8.

37. Dai J, Escara-Wilke J, Keller JM, Jung Y, Taichman RS, Pienta KJ, et al. Primary prostate cancer educates bone stroma through exosomal pyruvate kinase M2 to promote bone metastasis. J Exp Med. 2019;216(12):2883-99. 10.1084/jem.20190158.

38. Hu J, He Q, Tian T, Chang N, Qian L. Transmission of Exosomal TPX2 Promotes Metastasis and Resistance of NSCLC Cells to Docetaxel. Onco Targets Ther. 2023;16:197-210. 10.2147/ott.S401454.

39. Song J, Wang L, Ma Q, Yang Y, Yang Z, Wang B, et al. Let‑7i‑5p inhibits the proliferation and metastasis of colon cancer cells by targeting kallikrein‑related peptidase 6. Oncol Rep. 2018;40(3):1459-66. 10.3892/or.2018.6577.

40. Wu D-m, Deng S-h, Liu T, Han R, Zhang T, Xu Y. TGF-β-mediated exosomal lnc-MMP2-2 regulates migration and invasion of lung cancer cells to the vasculature by promoting MMP2 expression. Cancer Medicine. 2018;7(10):5118-29. <https://doi.org/10.1002/cam4.1758>.

41. Wang X, Li H, Lu X, Wen C, Huo Z, Shi M, et al. Melittin-induced long non-coding RNA NONHSAT105177 inhibits proliferation and migration of pancreatic ductal adenocarcinoma. Cell Death & Disease. 2018;9(10):940. 10.1038/s41419-018-0965-3.

42. Xu Y, Liu N, Wei Y, Zhou D, Lin R, Wang X, et al. Anticancer effects of miR-124 delivered by BM-MSC derived exosomes on cell proliferation, epithelial mesenchymal transition, and chemotherapy sensitivity of pancreatic cancer cells. Aging (Albany NY). 2020;12(19):19660-76. 10.18632/aging.103997.

43. Moirangthem A, Gondaliya P, Yan IK, Sayyed AA, Driscoll J, Patel T. Extracellular vesicle‑mediated miR‑126‑3p transfer contributes to inter‑cellular communication in the liver tumor microenvironment. Int J Oncol. 2023;62(2). 10.3892/ijo.2023.5479.

44. Bryant RJ, Pawlowski T, Catto JW, Marsden G, Vessella RL, Rhees B, et al. Changes in circulating microRNA levels associated with prostate cancer. Br J Cancer. 2012;106(4):768-74. 10.1038/bjc.2011.595.

45. Sheykhhasan M, Kalhor N, Sheikholeslami A, Dolati M, Amini E, Fazaeli H. Exosomes of Mesenchymal Stem Cells as a Proper Vehicle for Transfecting miR-145 into the Breast Cancer Cell Line and Its Effect on Metastasis. Biomed Res Int. 2021;2021:5516078. 10.1155/2021/5516078.

46. Li B-L, Lu W, Qu J-J, Ye L, Du G-Q, Wan X-P. Loss of exosomal miR-148b from cancer-associated fibroblasts promotes endometrial cancer cell invasion and cancer metastasis. Journal of Cellular Physiology. 2019;234(3):2943-53. <https://doi.org/10.1002/jcp.27111>.

47. Stiegelbauer V, Vychytilova-Faltejskova P, Karbiener M, Pehserl AM, Reicher A, Resel M, et al. miR-196b-5p Regulates Colorectal Cancer Cell Migration and Metastases through Interaction with HOXB7 and GALNT5. Clin Cancer Res. 2017;23(17):5255-66. 10.1158/1078-0432.Ccr-17-0023.

48. Rahmati S, Moeinafshar A, Rezaei N. The multifaceted role of extracellular vesicles (EVs) in colorectal cancer: metastasis, immune suppression, therapy resistance, and autophagy crosstalk. Journal of Translational Medicine. 2024;22(1):452. 10.1186/s12967-024-05267-8.

49. Gong W, Guo Y, Yuan H, Chai R, Wan Z, Zheng B, et al. Loss of exosomal miR-200b-3p from hypoxia cancer-associated fibroblasts promotes tumorigenesis and reduces sensitivity to 5-Flourouracil in colorectal cancer via upregulation of ZEB1 and E2F3. Cancer Gene Therapy. 2023;30(6):905-16. 10.1038/s41417-023-00591-5.

50. Han Q, Tan S, Gong L, Li G, Wu Q, Chen L, et al. Omental cancer-associated fibroblast-derived exosomes with low microRNA-29c-3p promote ovarian cancer peritoneal metastasis. Cancer Science. 2023;114(5):1929-42. <https://doi.org/10.1111/cas.15726>.

51. Huang W, Yan Y, Liu Y, Lin M, Ma J, Zhang W, et al. Exosomes with low miR-34c-3p expression promote invasion and migration of non-small cell lung cancer by upregulating integrin α2β1. Signal Transduction and Targeted Therapy. 2020;5(1):39. 10.1038/s41392-020-0133-y.
