# Supplementary material for: Extracellular vesicles in cancer´s communication: messages we can read and how to answer
Source: Mol Cancer. 2025 Mar 19;24:86. doi: 10.1186/s12943-025-02282-1 (PMC11921637; doi:10.1186/s12943-025-02282-1)
Supplement: Supplementary file 5 — Supplementary Material 5. [file 12943_2025_2282_MOESM5_ESM.docx]

**Table S5: EV-associated RNAs and proteins contributing to EV-mediated drug resistance**

| **Up-regulated in drug resistance** | | | | | |  |
| --- | --- | --- | --- | --- | --- | --- |
| *RNA* | *Tissue* | *Affected systems* | *Resistance to* | *In vitro/in vivo* | *Ref.* |  |
| circR-0002130 | Non-small cell lung cancer | ↓ miR-498 ↑ GLUT1, HK2, LDHA | osimertinib | In vitro, in vivo | [1] |  |
| lncR-ARSR | Renal cancer | ↓ miR-34, miR-449 ↑ AXL, c-MET | sunitinib | In vitro, in vivo | [2] |  |
| lncR-DILA1 | Breast cancer | ↑ cyclin D1 | tamoxifen | In vitro, in vivo | [3] |  |
| lncR-H19 | Colorectal cancer | ↓ miR-141 ↑ β-catenin | multidrug  resistance | In vitro, in vivo | [4] |  |
|  | Non-small cell lung cancer | n.s. | gefitinib | In vitro | [5] |  |
|  | various | | | | [6] (review article) |  |
| lncR-HOTAIR | Laryngeal cancer | ↓ miR-454-3p ↑ E2F2 | radiotherapy | In vitro, in vivo | [7] |  |
| lncR-IGFL2-AS1 | Renal cell carcinoma | ↑ TP53INP2 | sunitinib | In vitro, in vivo | [8] |  |
| lncR-POU3F3 | Esophageal squamous cell carcinoma | ↑ α-SMA ↑ fibroblast differentiation | cisplatin | In vitro | [9] |  |
| lncR-UCA1 | Non-small cell lung cancer | ↓ CDKN1A | gefitinib | In vitro, in vivo | [10] |  |
| lncR-VLDLR | Hepatocellular cancer | ↑ ABCG2 | sorafenib | In vitro | [11] |  |
| miR-9–5p, miR-195-5p, miR-203a-3p | Breast cancer | ↑ cancer stemness | docetaxel, doxorubicin | In vitro, in vivo | [12] |  |
| miR-223 | Gastric cancer | ↓ FBXW7 | doxorubicin | In vitro | [13] |  |
| miR-27a-3p | Glioblastoma | ↑ BTG2 | temozolomide | In vitro, in vivo | [14] |  |
| miR-3613-3p | Breast cancer | ↓ SOCS2 | *n.s.* | In vitro | [15] |  |
| miR-373 | Breast cancer | ↓ estrogen receptor | camptothecin | In vitro | [16] |  |
| **Up-regulated in drug resistance** - continued | | | | | | |
| *RNA* | | *Tissue* | *Affected systems* | *Resistance to* | *In vitro/in vivo* | *Ref.* |
| miR-378a-3p, miR-378d | Breast cancer | ↑ EZH2/STAT3 signaling, ↑ cancer stemness | doxorubicin, paclitaxel | In vitro, in vivo | [17] |  |
| miR-423-5p | Breast cancer | *n.s.* | cisplatin | In vitro | [18] |  |
| miR-429 | Ovarian cancer | ↓ CASR ↑ STAT3 | cisplatin | In vitro, in vivo | [19] |  |
| **Down-regulated in drug resistance** | | | | | |  |
| *RNA* | *Tissue* | *Affected systems* | *Resistance to* | *In vitro/in vivo* | *Ref.* |  |
| miR-7 | Non-small cell lung cancer | Reverses drug resistance via ↓ YAP | gefitinib | In vitro, in vivo | [20] |  |
| miR-30a-5p | Non-small cell lung cancer | Reverses drug resistance via ↓ PI3K/AKT pathway | gefitinib | In vitro, in vivo | [21] |  |
| **Proteins inducing drug resistance** | | | | | |  |
| *Protein* | *Tissue* | *Affected systems* | *Resistance to* | *In vitro/in vivo* | *Ref.* |  |
| ANXA6 | Triple-negative breast cancer | ↑ EGFR | gemcitabine | In vitro, ex vivo | [22] |  |
| TPX2 | Non-small cell lung cancer | ↑ WNT/β-catenin signaling pathway | docetaxel | In vitro, in vivo | [23] |  |

*n.s.: not specified*

1. Ma J, Qi G, Li L. A Novel Serum Exosomes-Based Biomarker hsa_circ_0002130 Facilitates Osimertinib-Resistance in Non-Small Cell Lung Cancer by Sponging miR-498. Onco Targets Ther. 2020;13:5293-307. 10.2147/ott.S243214.

2. Qu L, Ding J, Chen C, Wu ZJ, Liu B, Gao Y, et al. Exosome-Transmitted lncARSR Promotes Sunitinib Resistance in Renal Cancer by Acting as a Competing Endogenous RNA. Cancer Cell. 2016;29(5):653-68. 10.1016/j.ccell.2016.03.004.

3. Shi Q, Li Y, Li S, Jin L, Lai H, Wu Y, et al. LncRNA DILA1 inhibits Cyclin D1 degradation and contributes to tamoxifen resistance in breast cancer. Nature Communications. 2020;11(1):5513. 10.1038/s41467-020-19349-w.

4. Ren J, Ding L, Zhang D, Shi G, Xu Q, Shen S, et al. Carcinoma-associated fibroblasts promote the stemness and chemoresistance of colorectal cancer by transferring exosomal lncRNA H19. Theranostics. 2018;8(14):3932-48. 10.7150/thno.25541.

5. Lei Y, Guo W, Chen B, Chen L, Gong J, Li W. Tumor‑released lncRNA H19 promotes gefitinib resistance via packaging into exosomes in non‑small cell lung cancer. Oncol Rep. 2018;40(6):3438-46. 10.3892/or.2018.6762.

6. Shermane Lim YW, Xiang X, Garg M, Le MTN, Li-Ann Wong A, Wang L, et al. The double-edged sword of H19 lncRNA: Insights into cancer therapy. Cancer Letters. 2021;500:253-62. <https://doi.org/10.1016/j.canlet.2020.11.006>.

7. Cui X, Xiao D, Cui Y, Wang X. Exosomes-Derived Long Non-Coding RNA HOTAIR Reduces Laryngeal Cancer Radiosensitivity by Regulating microRNA-454-3p/E2F2 Axis. Onco Targets Ther. 2019;12:10827-39. 10.2147/ott.S224881.

8. Pan Y, Lu X, Shu G, Cen J, Lu J, Zhou M, et al. Extracellular Vesicle-Mediated Transfer of LncRNA IGFL2-AS1 Confers Sunitinib Resistance in Renal Cell Carcinoma. Cancer Res. 2023;83(1):103-16. 10.1158/0008-5472.Can-21-3432.

9. Tong Y, Yang L, Yu C, Zhu W, Zhou X, Xiong Y, et al. Tumor-Secreted Exosomal lncRNA POU3F3 Promotes Cisplatin Resistance in ESCC by Inducing Fibroblast Differentiation into CAFs. Mol Ther Oncolytics. 2020;18:1-13. 10.1016/j.omto.2020.05.014.

10. Xu T, Yan S, Wang M, Jiang L, Ma P, Lu B, et al. LncRNA UCA1 Induces Acquired Resistance to Gefitinib by Epigenetically Silencing CDKN1A Expression in Non-small-Cell Lung Cancer. Front Oncol. 2020;10:656. 10.3389/fonc.2020.00656.

11. Takahashi K, Yan IK, Wood J, Haga H, Patel T. Involvement of extracellular vesicle long noncoding RNA (linc-VLDLR) in tumor cell responses to chemotherapy. Mol Cancer Res. 2014;12(10):1377-87. 10.1158/1541-7786.Mcr-13-0636.

12. Shen M, Dong C, Ruan X, Yan W, Cao M, Pizzo D, et al. Chemotherapy-Induced Extracellular Vesicle miRNAs Promote Breast Cancer Stemness by Targeting ONECUT2. Cancer Res. 2019;79(14):3608-21. 10.1158/0008-5472.Can-18-4055.

13. Gao H, Ma J, Cheng Y, Zheng P. Exosomal Transfer of Macrophage-Derived miR-223 Confers Doxorubicin Resistance in Gastric Cancer. Onco Targets Ther. 2020;13:12169-79. 10.2147/ott.S283542.

14. Chen L, Li Z, Hu S, Deng Q, Hao P, Guo S. Extracellular vesicles carry miR-27a-3p to promote drug resistance of glioblastoma to temozolomide by targeting BTG2. Cancer Chemotherapy and Pharmacology. 2022;89(2):217-29. 10.1007/s00280-021-04392-1.

15. Liu Y, Yang Y, Du J, Lin D, Li F. MiR-3613-3p from carcinoma-associated fibroblasts exosomes promoted breast cancer cell proliferation and metastasis by regulating SOCS2 expression. IUBMB Life. 2020;72(8):1705-14. 10.1002/iub.2292.

16. Eichelser C, Stückrath I, Müller V, Milde-Langosch K, Wikman H, Pantel K, et al. Increased serum levels of circulating exosomal microRNA-373 in receptor-negative breast cancer patients. Oncotarget. 2014;5(20):9650-63. 10.18632/oncotarget.2520.

17. Yang Q, Zhao S, Shi Z, Cao L, Liu J, Pan T, et al. Chemotherapy-elicited exosomal miR-378a-3p and miR-378d promote breast cancer stemness and chemoresistance via the activation of EZH2/STAT3 signaling. Journal of Experimental & Clinical Cancer Research. 2021;40(1):120. 10.1186/s13046-021-01901-1.

18. Wang B, Zhang Y, Ye M, Wu J, Ma L, Chen H. Cisplatin-resistant MDA-MB-231 Cell-derived Exosomes Increase the Resistance of Recipient Cells in an Exosomal miR-423-5p-dependent Manner. Curr Drug Metab. 2019;20(10):804-14. 10.2174/1389200220666190819151946.

19. Li T, Lin L, Liu Q, Gao W, Chen L, Sha C, et al. Exosomal transfer of miR-429 confers chemoresistance in epithelial ovarian cancer. Am J Cancer Res. 2021;11(5):2124-41.

20. Chen R, Qian Z, Xu X, Zhang C, Niu Y, Wang Z, et al. Exosomes-transmitted miR-7 reverses gefitinib resistance by targeting YAP in non-small-cell lung cancer. Pharmacol Res. 2021;165:105442. 10.1016/j.phrs.2021.105442.

21. Wang F, Meng F, Wong SCC, Cho WCS, Yang S, Chan LWC. Combination therapy of gefitinib and miR-30a-5p may overcome acquired drug resistance through regulating the PI3K/AKT pathway in non-small cell lung cancer. Therapeutic Advances in Respiratory Disease. 2020;14:1753466620915156. 10.1177/1753466620915156.

22. Li T, Tao Z, Zhu Y, Liu X, Wang L, Du Y, et al. Exosomal annexin A6 induces gemcitabine resistance by inhibiting ubiquitination and degradation of EGFR in triple-negative breast cancer. Cell Death Dis. 2021;12(7):684. 10.1038/s41419-021-03963-7.

23. Hu J, He Q, Tian T, Chang N, Qian L. Transmission of Exosomal TPX2 Promotes Metastasis and Resistance of NSCLC Cells to Docetaxel. Onco Targets Ther. 2023;16:197-210. 10.2147/ott.S401454.
