# Supplementary material for: Extracellular vesicles in cancer´s communication: messages we can read and how to answer
Source: Mol Cancer. 2025 Mar 19;24:86. doi: 10.1186/s12943-025-02282-1 (PMC11921637; doi:10.1186/s12943-025-02282-1)
Supplement: Supplementary file 6 — Supplementary Material 6. [file 12943_2025_2282_MOESM6_ESM.docx]

**Table S6: Recent and current clinical trials exploring the diagnostic and therapeutic potential of exosomes**

| **Exosomes as the source of biomarkers** | | | | | |
| --- | --- | --- | --- | --- | --- |
| *Tissue* | *Code* | *Sample* | *Markers/class* | *Type of biomarker* | *Ref.* |
| Bladder cancer | NCT05270174 | Urine | lncRNA-ELNAT1 | Metastatic | [1] |
|  | NCT06193941 | Urine | RNA^1^ | Diagnostic | [2] |
| Breast cancer | NCT05798338 | Plasma | protein^1^ | Stage | [3] |
|  | NCT05286684 | Cerebrospinal fluid | protein^1^ | Metastatic | [4] |
| Colorectal cancer | NCT04394572 | Blood | macromolecules^1^, integrins^1^, proteases^1^ | Diagnostic | [5] |
|  | NCT06342401 | Blood | miRNA^1^ | Diagnostic | [6] |
| Esophageal cancer  & gastric cancer | NCT06278064 | Plasma | protein^1^ | Diagnostic, prognostic | [7] |
| Gastric cancer | NCT06023121 | *n.s.* | *n.s.* | Diagnostic | [8] |
|  | NCT06342427 | Serum | miRNA^1^ | Diagnostic | [9] |
| Glioma | NCT06116903 | Blood | Nucleic acids^1^ | Diagnostic | [10] |
| Intrahepatic cholangiocarcinoma | NCT06381648 | Blood | miRNA^1^ | Metastatic | [11] |
| Liver cancer | NCT06342414 | Blood | miRNA^1^ | Diagnostic | [12] |
| Lung cancer | NCT03830619 | Serum | lncRNA^1^ | Diagnostic | [13] |
|  | NCT06026735 | Blood, cerebrospinal fluid | cell-related substances^1^ | Metastatic, response to treatment | [14] |
| Meningioma | NCT06104930 | Blood | exosome level, DNA methylation^1^ | Progression | [15] |
| Non-small cell lung cancer | NCT05218759 | Blood | miRNA^1^ | Response to treatment (anlotinib) | [16] |
| Oral squamous cell carcinoma | NCT06469892 | Saliva, plasma | miR-185 | Diagnostic | [17] |
| Oropharyngeal squamous cell carcinoma | NCT02147418 | Oropharyngeal rinse | protein^1^ | Diagnostic | [18] |

^1^... study aims to find new biomarker molecules in the stated class of molecules; *n.s.* = not specified

| **EVs as the source of biomarkers** - continued | | | | | |
| --- | --- | --- | --- | --- | --- |
| *Tissue* | *Code* | *Sample* | *Markers/class* | *Type of biomarker* | *Ref.* |
| Osteosarcoma | NCT05101655 | Plasma | exosome level, subgroups | Metastatic | [19] |
|  | NCT03108677 | Blood | RNA^1^ | Metastatic | [20] |
| Ovarian cancer | NCT06558019 | *n.s.* | *n.s.* (OCS diagnostic kit)^2^ | Recurrence | [21] |
| Pancreatic cancer | NCT06108531 | Blood | protein^1^ | Diagnostic | [22] |
| Pancreatic ductal adenocarcinoma | NCT05625529 | Blood | proteins (ExoVerita™)^2^ | Diagnostic | [23] |
|  | NCT06388967 | Plasma | 13 different miRNA | Diagnostic | [24] |
| Prostate cancer | NCT04720599 | Urine | PCA3, ERG, and SPDEF gene expression (ExoDx™ Prostate test)^2^ | Diagnostic | [25] |
|  | NCT06326216 | Urine, blood | exosome level (cancer-derived) | Response to treatment (surgery) | [26] |
| Rectal cancer | NCT04852653 | Blood | tumor DNA, protein^1^ | Response to treatment (neoadjuvant chemotherapy) | [27] |
|  | NCT03874559 | Serum | exosome level, *n.s.* | Response to treatment (neoadjuvant chemotherapy) | [28] |
| Renal carcinoma | NCT04053855 | Urine | exosome level  (CD9^+^ /CA9^+^) | Diagnostic | [29] |
| Sarcoma | NCT03800121 | Blood | exosome level, protein^1^, RNA^1^ | Disease state, recurrence, response to treatment | [30] |
| Thyroid cancer | NCT05463107 | Urine | 11 proteins | Diagnostic | [31] |
|  | NCT04948437 | Urine | thyroglobulin, galectin-3 | Follow-up after treatment (surgery, radiotherapy) | [32] |
|  | NCT03488134 | Urine | thyroglobulin, galectin-3 | Prognostic, recurrence | [33] |

^1^... study aims to find new biomarker molecules in the stated class of molecules; ^2^... study aims to validate the performance of a commercial diagnostic kit (specified in brackets); *n.s.* = not specified

| **EVs as the carriers and markers of resistance to treatment** | | | | | |
| --- | --- | --- | --- | --- | --- |
| *Tissue* | *Code* | *Sample* | *Markers/class* | *Goal of the study* | *Ref.* |
| Non-Hodgkin  B-cell lymphoma | NCT03985696 | Plasma | CD20, PD-L1 | Investigation of EVs‘ role  in immunotherapy escape | [34] |
| **EV-based vaccines in cancer** | | | | | |
| *Tissue* | *Code* | *EV origin* | *Loaded cargo* | *Goal of the study* | *Ref.* |
| Lung cancer | NCT01159288 | dendritic cells | tumor antigen proteins | Innate and adaptive immunity activation | [35] |
| **EVs as treatment of cancer or associated pathologies** | | | | | |
| *Tissue* | *Code* | *EVs* | *Intervention* | *Goal of the study* | *Ref.* |
| Acute myeloid leukemia | NCT06245746 | Umbilical cord-derived mesenchymal stem cells | Intravenous administration | Reversing myelosuppression | [36] |
| Colon cancer | NCT01294072 | Plant; loaded with curcumin | Dietary supplementation | Inhibition of cancer cell growth | [37] |
| Head and neck cancer | NCT01668849 | Grape | Dietary supplementation | Reduce the incidence of oral mucositis during radiation and chemotherapy | [38] |
| Pancreatic ductal adenocarcinoma (with KrasG12D mutation) | NCT03608631 | Mesenchymal  stromal cells loaded with siRNA against KrasG12D | Intravenous administration | Cancer treatment | [39] |
| Rectal cancer | NCT06536712 | Human placenta mesenchymal  stem cells | Intraperitoneal administration after surgery | Prevent anastomosis leaks after low anterior resection | [40] |

1. ClinicalTrials.gov [Internet]. Bethesda (MD): National Library of Medicine (US); 2000 -. Identifier NCT05270174, A Prospective, Multicenter Cohort Study of Urinary Exosome lncRNAs for Preoperative Diagnosis of Lymphatic Metastasis in Patients With Bladder Cancer; 2022/02/09 [cited 2025/02/11]. Available from: <https://clinicaltrials.gov/study/NCT05270174>.

2. ClinicalTrials.gov [Internet]. Bethesda (MD): National Library of Medicine (US); 2000 -. Identifier NCT06193941, Research on the Accurate Diagnosis of Urinary Tract Tumors and the Development of Kits; 2023/12/21 [cited 2025/02/11]. Available from: <https://clinicaltrials.gov/study/NCT06193941>.

3. ClinicalTrials.gov [Internet]. Bethesda (MD): National Library of Medicine (US); 2000 -. Identifier NCT05798338, Characterization of Extracellular Vesicles in Breast Cancer Patients' Plasma by Single Molecule Detection Array (SiMoA) Digital ELISA; 2023/03/21 [cited 2025/02/11]. Available from: <https://clinicaltrials.gov/study/NCT05798338>.

4. ClinicalTrials.gov [Internet]. Bethesda (MD): National Library of Medicine (US); 2000 -. Identifier NCT05286684, Feasibility of Exosome Analysis in Cerebrospinal Fluid During the Diagnostic Workup of Metastatic Meningitis from Breast Cancer; 2022/01/31 [cited 2025/02/11]. Available from: <https://clinicaltrials.gov/study/NCT05286684>.

5. ClinicalTrials.gov [Internet]. Bethesda (MD): National Library of Medicine (US); 2000 -. Identifier NCT04394572, Identification of New Diagnostic Protein Markers for Colorectal Cancer in Circulating Tumor Exosomes; 2020/01/31 [cited 2025/02/11]. Available from: <https://clinicaltrials.gov/study/NCT04394572>.

6. ClinicalTrials.gov [Internet]. Bethesda (MD): National Library of Medicine (US); 2000 -. Identifier NCT06342401, Development and Validation fo an Exosome-Based and Machine Learning Powered Liquid Biopsy for the Detection of Early-Onset Colorectal Cancer; 2024/03/26 [cited 2025/02/11]. Available from: <https://clinicaltrials.gov/study/NCT06342401>.

7. ClinicalTrials.gov [Internet]. Bethesda (MD): National Library of Medicine (US); 2000 -. Identifier NCT06278064, Plasma Extracellular Vesicle Quantitative Proteomic Analysis for Early Diagnosis of Upper Gastrointestinal Cancers; 2024/02/19 [cited 2025/02/11]. Available from: <https://clinicaltrials.gov/study/NCT06278064>.

8. ClinicalTrials.gov [Internet]. Bethesda (MD): National Library of Medicine (US); 2000 -. Identifier NCT06023121, Use of a Liquid Biopsy Signature as Blood Biomarker for Early Detection and Monitoring Early-onset Gastric Cancer; 2023/08/29 [cited 2025/02/11]. Available from: <https://clinicaltrials.gov/study/NCT06023121>.

9. ClinicalTrials.gov [Internet]. Bethesda (MD): National Library of Medicine (US); 2000 -. Identifier NCT06342427, Early Detection of Stomach Cancer With a Liquid Biopsy Based on Exosomal Micro-RNA; 2024/03/26 [cited 2025/02/11]. Available from: <https://clinicaltrials.gov/study/NCT06342427>.

10. ClinicalTrials.gov [Internet]. Bethesda (MD): National Library of Medicine (US); 2000 -. Identifier NCT06116903, Clinical Relevance of Detecting Molecular Abnormalities in Glial Tumor Exosomes; 2023/10/31 [cited 2025/02/11]. Available from: <https://clinicaltrials.gov/study/NCT06116903>.

11. ClinicalTrials.gov [Internet]. Bethesda (MD): National Library of Medicine (US); 2000 -. Identifier NCT06381648, An Exosome-based Liquid Biopsy Signature for Pre-operative Identification of Lymph Node Metastasis in Patients With Intrahepatic Cholangiocarcinoma; 2024/04/19 [cited 2025/02/11]. Available from: <https://clinicaltrials.gov/study/NCT06381648>.

12. ClinicalTrials.gov [Internet]. Bethesda (MD): National Library of Medicine (US); 2000 -. Identifier NCT06342414, An Exosome-Based Liquid Biopsy for the Differential Diagnosis of Primary Liver Cancer; 2024/03/26 [cited 2025/02/11]. Available from: <https://clinicaltrials.gov/study/NCT06342414>.

13. ClinicalTrials.gov [Internet]. Bethesda (MD): National Library of Medicine (US); 2000 -. Identifier NCT03830619, Serum Exosomal Long Noncoding RNAs as Potential Biomarkers for Lung Cancer Diagnosis; 2019/01/24 [cited 2025/02/11]. Available from: <https://clinicaltrials.gov/study/NCT03830619>.

14. ClinicalTrials.gov [Internet]. Bethesda (MD): National Library of Medicine (US); 2000 -. Identifier NCT06026735, Comprehensive Profiling of Promising Biomarkers in Exosomes From Non-small Cell Lung Cancer With Central Nervous System Metastasis; 2022/10/25 [cited 2025/02/11]. Available from: <https://clinicaltrials.gov/study/NCT06026735>.

15. ClinicalTrials.gov [Internet]. Bethesda (MD): National Library of Medicine (US); 2000 -. Identifier NCT06104930, Plasma Extracellular Vesicles in Meningioma Patients Following Radiotherapy as Liquid Biopsy; 2023/10/11 [cited 2025/02/11]. Available from: <https://clinicaltrials.gov/study/NCT06104930>.

16. ClinicalTrials.gov [Internet]. Bethesda (MD): National Library of Medicine (US); 2000 -. Identifier NCT05218759, Exosomes Detection for the Prediction of the Efficacy and Adverse Reactions of Anlotinib in Patients With Advanced NSCLC; 2022/01/19 [cited 2025/02/11]. Available from: <https://clinicaltrials.gov/study/NCT05218759>.

17. ClinicalTrials.gov [Internet]. Bethesda (MD): National Library of Medicine (US); 2000 -. Identifier NCT06469892, Application of Salivary and Plasma Exosomes in the Diagnosis of Oral Leukoplakia Malignant Transformation and Prognosis Monitoring of Oral Cancer; 2024/06/14 [cited 2025/02/11]. Available from: <https://clinicaltrials.gov/study/NCT06469892>.

18. ClinicalTrials.gov [Internet]. Bethesda (MD): National Library of Medicine (US); 2000 -. Identifier NCT02147418, An Observational, Single-Institution Pilot/Feasibility Study of Exosome Testing as a Screening Modality for Human Papillomavirus-Positive Oropharyngeal Squamous Cell Carcinoma; 2014/05/22 [cited 2025/02/11]. Available from: <https://clinicaltrials.gov/study/NCT02147418>.

19. ClinicalTrials.gov [Internet]. Bethesda (MD): National Library of Medicine (US); 2000 -. Identifier NCT05101655, Construction and Clinical Application of Microfluidic Exosome Chip for Early Diagnosis of Pulmonary Metastasis of Osteosarcoma; 2021/10/07 [cited 2025/02/11]. Available from: <https://clinicaltrials.gov/study/NCT05101655>.

20. ClinicalTrials.gov [Internet]. Bethesda (MD): National Library of Medicine (US); 2000 -. Identifier NCT03108677, A Pilot Study of Circulating Exosome RNA as Diagnostic and Prognostic Markers in Lung Metastases of Primary High-Grade Osteosarcoma; 2017/04/25 [cited 2025/02/11]. Available from: <https://clinicaltrials.gov/study/NCT03108677>.

21. ClinicalTrials.gov [Internet]. Bethesda (MD): National Library of Medicine (US); 2000 -. Identifier NCT06558019, Exosome-based OCS Scores for Predicting Ovarian Cancer Recurrence: a Prospective Multicenter Observational Cohort; 2024/07/22 [cited 2025/02/11]. Available from: <https://clinicaltrials.gov/study/NCT06558019>.

22. ClinicalTrials.gov [Internet]. Bethesda (MD): National Library of Medicine (US); 2000 -. Identifier NCT06108531, Study on the Application of Tumor Exosome Liquid Biopsy in the Early Diagnosis of Pancreatic Cancer; 2023/10/23 [cited 2025/02/11]. Available from: <https://clinicaltrials.gov/study/NCT06108531>.

23. ClinicalTrials.gov [Internet]. Bethesda (MD): National Library of Medicine (US); 2000 -. Identifier NCT05625529, Exoluminate Study: Observational Registry Study to Assess Exo-PDAC Assay Performance for Detection of Pancreatic Adenocarcinoma (PDAC) in High-Risk or Clinically Suspicious Patients; 2022/11/15 [cited 2025/02/11]. Available from: <https://clinicaltrials.gov/study/NCT05625529>.

24. ClinicalTrials.gov [Internet]. Bethesda (MD): National Library of Medicine (US); 2000 -. Identifier NCT06388967, Early Detection of Pancreatic Cancer: Prospective Study; 2024/04/19 [cited 2025/02/11]. Available from: <https://clinicaltrials.gov/study/NCT06388967>.

25. ClinicalTrials.gov [Internet]. Bethesda (MD): National Library of Medicine (US); 2000 -. Identifier NCT04720599, Clinical Evaluation of ExoDx™ Prostate(IntelliScore) in Men Presenting for Initial Prostate Biopsy; 2021/01/20 [cited 2025/02/11]. Available from: <https://clinicaltrials.gov/study/NCT04720599>.

26. ClinicalTrials.gov [Internet]. Bethesda (MD): National Library of Medicine (US); 2000 -. Identifier NCT06326216, Determination of Baseline Levels for Prostate Cancer-Derived Extracellular Vesicles Following Local Treatment of Prostate Cancer; 2024/03/15 [cited 2025/02/11]. Available from: <https://clinicaltrials.gov/study/NCT06326216>.

27. ClinicalTrials.gov [Internet]. Bethesda (MD): National Library of Medicine (US); 2000 -. Identifier NCT04852653, A Prospective Feasibility Study Evaluating Extracellular Vesicles Obtained by Liquid Biopsy for Neoadjuvant Treatment Response Assessment in Rectal Cancer; 2021/04/16 [cited 2025/02/11]. Available from: <https://clinicaltrials.gov/study/NCT04852653>.

28. ClinicalTrials.gov [Internet]. Bethesda (MD): National Library of Medicine (US); 2000 -. Identifier NCT03874559, Exosomal As Correlative Biomarker in Clinical Outcomes in Patients Undergoing Neoadjuvant Chemoradiation Therapy for Rectal Cancer; 2019/03/11 [cited 2025/02/11]. Available from: <https://clinicaltrials.gov/study/NCT03874559>.

29. ClinicalTrials.gov [Internet]. Bethesda (MD): National Library of Medicine (US); 2000 -. Identifier NCT04053855, Evaluation of Urinary Exosomes Presence From Clear Cell Renal Cell Carcinoma; 2019/08/09 [cited 2025/02/11]. Available from: <https://clinicaltrials.gov/study/NCT04053855>.

30. ClinicalTrials.gov [Internet]. Bethesda (MD): National Library of Medicine (US); 2000 -. Identifier NCT03800121, Study of Exosomes in Monitoring Patients With Sarcoma (EXOSARC); 2019/01/03 [cited 2025/02/11]. Available from: <https://clinicaltrials.gov/study/NCT03800121>.

31. ClinicalTrials.gov [Internet]. Bethesda (MD): National Library of Medicine (US); 2000 -. Identifier NCT05463107, Correlation Between Various Urinary Exosomal Protein Biomarkers and Pathological Manifestation in Thyroid Follicular Neoplasm: Early and Pre-operative Diagnosis of Follicular Thyroid Cancer; 2022/07/14 [cited 2025/02/11]. Available from: <https://clinicaltrials.gov/study/NCT05463107>.

32. ClinicalTrials.gov [Internet]. Bethesda (MD): National Library of Medicine (US); 2000 -. Identifier NCT04948437, Urinary Exosomal Biomarkers of Thyroglobulin and Galectin-3 for Prognosis and Follow-up in Patients of Well-differentiated Thyroid Cancer; 2021/06/24 [cited 2025/02/11]. Available from: <https://clinicaltrials.gov/study/NCT04948437>.

33. ClinicalTrials.gov [Internet]. Bethesda (MD): National Library of Medicine (US); 2000 -. Identifier NCT03488134, A Prospective Study of Predicting Prognosis and Recurrence of Thyroid Cancer Via New Biomarkers, Urinary Exosomal Thyroglobulin and Galectin-3; 2018/03/28 [cited 2025/02/11]. Available from: <https://clinicaltrials.gov/study/NCT03488134>.

34. ClinicalTrials.gov [Internet]. Bethesda (MD): National Library of Medicine (US); 2000 -. Identifier NCT03985696, Exosomes and Resistance to Immunotherapy in Aggressive Non-Hodgkin B-cell Lymphomas (B-NHL); 2019/06/11 [cited 2025/02/11]. Available from: <https://clinicaltrials.gov/study/NCT03985696>.

35. ClinicalTrials.gov [Internet]. Bethesda (MD): National Library of Medicine (US); 2000 -. Identifier NCT01159288, Phase II Trial of a Vaccination With Tumor Antigen-loaded Dendritic Cell-derived Exosomes on Patients With Unresectable Non Small Cell Lung Cancer Responding to Induction Chemotherapy; 2010/07/08 [cited 2025/02/11]. Available from: <https://clinicaltrials.gov/study/NCT01159288>.

36. ClinicalTrials.gov [Internet]. Bethesda (MD): National Library of Medicine (US); 2000 -. Identifier NCT06245746, A Single-center, Prospective Trial of the Safety and Efficacy of UCMSC-Exo in Consolidation Chemotherapy-induced Myelosuppression in Patients With Acute Myeloid Leukemia After Achieving Complete Remission; 2024/01/30 [cited 2025/02/11]. Available from: <https://clinicaltrials.gov/study/NCT06245746>.

37. ClinicalTrials.gov [Internet]. Bethesda (MD): National Library of Medicine (US); 2000 -. Identifier NCT01294072, Phase I Clinical Trial Investigating the Ability of Plant Exosomes to Deliver Curcumin to Normal and Malignant Colon Tissue; 2011/02/03 [cited 2025/02/11]. Available from: <https://clinicaltrials.gov/study/NCT01294072>.

38. ClinicalTrials.gov [Internet]. Bethesda (MD): National Library of Medicine (US); 2000 -. Identifier NCT01668849, Preliminary Clinical Trial Investigating the Ability of Plant Exosomes to Abrogate Oral Mucositis Induced by Combined Chemotherapy and Radiation in Head and Neck Cancer Patients; 2012/08/06 [cited 2025/02/11]. Available from: <https://clinicaltrials.gov/study/NCT01668849>.

39. ClinicalTrials.gov [Internet]. Bethesda (MD): National Library of Medicine (US); 2000 -. Identifier NCT03608631, Phase I Study of Mesenchymal Stromal Cells-Derived Exosomes with KrasG12D SiRNA for Metastatic Pancreas Cancer Patients Harboring KrasG12D Mutation; 2018/07/16 [cited 2025/02/11]. Available from: <https://clinicaltrials.gov/study/NCT03608631>.

40. ClinicalTrials.gov [Internet]. Bethesda (MD): National Library of Medicine (US); 2000 -. Identifier NCT06536712, Investigating the Effect of Intraperitoneal Administration of Exosome in Preventing Early Anastomotic Leakage in Rectal Cancer Patients Who Undergo Low Anterior Resection; 2024/07/20 [cited 2025/02/11]. Available from: <https://clinicaltrials.gov/study/NCT06536712>.
